# Discontinuing antidepressant medication: a qualitative evidence synthesis and logic model based on health professionals’ views

**DOI:** 10.1186/s12913-025-13445-7

**Published:** 2025-09-30

**Authors:** Loukia Christoforou, Katy Sutcliffe

**Affiliations:** 1 Nicosia, Cyprus; 2 Institute of Education, EPPI Centre, UCL Social Research Institute, University College London, 10 Woburn Square, London, WC1H 0NR UK

**Keywords:** Depression, Antidepressants, Health professionals, Discontinuation, Systematic review, Qualitative, Social ecological model, Logic model

## Abstract

**Background:**

Increased long-term antidepressant medication (ADM) use can lead to serious psychological and physical public health implications, as well as increased financial costs and social injustice. Given that health professionals (HPs) can influence decisions on ADM discontinuation, it is imperative that to promote and enable discontinuation when the medication is no longer indicated, their perspectives around this issue are explored in depth. This systematic review of qualitative evidence aimed to identify HPs’ perspectives on the barriers and facilitators to ADM discontinuation / deprescribing.

**Methods:**

Eligible studies were primary studies, on HPs’ views on ADM discontinuation / deprescribing, that used qualitative data collection and analysis, and were published in English. We searched PubMed, CINAHL, PsycINFO, the British Journal of General Practice, the Networked Digital Library of Theses and Dissertations, and Google Scholar, from July 2018 until May 2022, and harvested studies from a review published in 2019. The PubMed search was repeated post-synthesis, in January 2024, to identify and incorporate studies published since May 2022. Data was synthesised using thematic synthesis and further analysed using Bronfenbrenner’s Social Ecological Model (SEM) and a logic model. One reviewer extracted data and assessed study quality, which was checked by a second reviewer. Coding and development of themes and sub-themes was undertaken by one reviewer and further developed through discussion with a second reviewer.

**Results:**

Fourteen studies were included capturing the views of over 280 HPs. The synthesis yielded eight analytical themes: Perceptions of ADM; Perceptions of Depression; Sense of Professional Duty; Confidence in Supporting ADM Discontinuation; Assessment of Patients’ Circumstances and Characteristics; Assessment of Patients’ Desires, Motivations and Capabilities; Systemic Healthcare Delivery Issues; and, Societal Norms and Pressures.

**Conclusions:**

Use of the SEM and development of a logic model uncovered possible pathways and underlying reasons through which factors influencing ADM discontinuation interrelate both within and across societal levels. Societal norms and pressures, and systemic healthcare delivery issues, appear to influence directly or indirectly all aspects around ADM discontinuation / deprescribing. Future research and interventions could target barriers highlighted by these two themes demonstrating the largest impact.

**Supplementary Information:**

The online version contains supplementary material available at 10.1186/s12913-025-13445-7.

## Background

Antidepressant medication (ADM) is primarily indicated for clinical depression although it is also approved by the Food and Drug Administration for other conditions including generalised anxiety disorder, obsessive compulsive disorder, post-traumatic stress disorder and chronic pain [1, 2]. Since the 1980s, ADM prescriptions have risen steadily in many Western countries [3]. In the UK, between 2015 and 2019, there was a 25% increase in the annual number of the ten most prescribed ADMs [4]. Between 2017 and 2018, it was estimated that 17% of UK’s adult population received one or more ADM prescriptions [5].

Studies have shown that ADM, especially when combined with psychotherapy or psychiatric consult, can provide symptom relief of depression and / or anxiety mental health conditions and prevent them from coming back [6–10]. However, despite the well-documented increase in ADM prescribing, data suggests no corresponding increase in depression incidence [11–14]. A leading influencing factor is the increased ADM treatment duration with prescribing guidelines shifting towards longer-term maintenance treatment with average duration over two years [12, 15, 16]. While maintenance treatment may be indicated for some, for a considerable percentage of patients (30–50%), long-term use is not supported by evidence [17]. Recent study findings suggest that almost half of people on long-term ADM treatment can discontinue the medication without relapsing [18].

Non-indicated long-term ADM use may increase risks of adverse effects which are often underreported in clinical trials [19, 20], the severity of which vary within and across different ADM types [21–23]. Between 2001 and 2011 in the US, hospital admissions due to ADM-related adverse effects noted a 17.6% increase for 18–64 years old age groups and a 64.8% increase for 65 years or older age groups [24]. While some side-effects appear soon after treatment initiation and are described as mild and short-term, others may last longer and continue even after ADM is discontinued [25]. Serious side-effects include gastrointestinal bleeding, weight gain, sleep disturbance, and affective disturbances such as emotional blunting [26], loss of personal agency [27] and suicidality [28]. Long-term ADM use among older people has shown to expose patients to increased risk of falls, fractures, hyponatraemia, strokes and death [29].

In addition to side-effects, ADM may cause withdrawal symptoms which are experienced by approximately half of patients who abruptly stop or gradually taper the dose [30, 31]. Withdrawal symptoms have been recognised as more severe and long-lasting than what was previously thought [32, 33]. The most common include gastrointestinal upset, dizziness, lethargy, anxiety, dysphoria, sleep disturbance and headache [34, 35]. Paraesthesia (or ‘brain zaps’), panic, and increased depressive and suicidal symptoms are among the more severe ones [36–39]. Long-term ADM users are more likely to experience withdrawal symptoms, which can lead them to continue seeking ADM and decrease their chances for successful discontinuation in the future [40–42].

Long-term ADM use may also result in higher financial costs. Contrary to older findings [43], it is suggested that ADM treatment for persistent depression or anxiety is not associated with reduction in costs relating to these disorders and is even found to contribute to increased healthcare and patient costs [44].

Initiation and long-term ADM use is often justified by professionals [45], researchers [46–51], and the general public [52, 53], on the hypothesis that depression relates to brain-based chemical imbalances / abnormalities, particularly referring to serotonin. However, the exact cause of depression - and, consequently, the mechanism through which ADM may exert therapeutic effects - remains heavily debated. A recent umbrella review that challenged the serotonin hypothesis of depression when it concluded that there is “no convincing evidence that depression is associated with, or caused by, lower serotonin concentrations or activity” [54], p. 11, prompted notable academic controversy [55, 56]. Other studies suggest that ADM is only marginally efficacious compared to placebos, that publication bias inflates its apparent efficacy, and that lack of reporting transparency in real-world studies suggest lower ADM effectiveness than reported [57–61].

The perception that depression can be solely caused by chemical abnormalities can have social justice implications as it poses a risk of medicalising understandable reactions to life circumstances which are often outside of peoples’ control and are arguably most frequently experienced by marginalised communities, racial minorities, or women [62–66].

Given the range of possible negative physical and psychological effects of ADM, its uncertain efficacy / effectiveness, as well as potential social justice implications, overdiagnosis and overtreatment with ADM constitutes a public health concern. Promoting ADM discontinuation / deprescribing when the medication is no longer indicated is considered as an important step in addressing these concerns.

Given that HPs are often viewed by patients as authoritative figures [67] due to their recognised knowledge, experience, expertise and legitimate position [68], they are likely to influence and shape decisions around ADM discontinuation. Due to this power-dynamic and the complexity characterising decision-making around this issue, there is a need to explore the perspectives of HPs around ADM discontinuation.

Existing evidence syntheses including HPs’ perspectives on deprescribing have predominantly focused on psychotropic medications more generally, other drug classes, or on medication overall, with comparatively less attention to ADM specifically (e.g [69–74]). A recent qualitative systematic review on ADM discontinuation which sought to synthesise studies focusing on the perspectives of both HPs and patients, did not synthesise HPs’ perspectives in the end due to ‘thin’ data [75]. The authors noted the need for further research from HPs perspectives including, but not limited to, that of GPs. This study therefore aimed to fill this gap in knowledge by exploring HPs’ perspectives on the barriers and facilitators to ADM discontinuation / deprescribing, using the systematic review (SR) methodology to conduct a synthesis of qualitative evidence.

## Methods

### Conceptual framework and inclusion criteria

To be included in the review, studies had to: (a) use qualitative data collection and analysis; (b) present data on HPs’ perspectives / views about deprescribing or helping patients discontinue ADM; and (c) be published in English. Mixed-method studies were eligible if they included a qualitative aspect which could be separately extracted.

ADM was defined as any medication falling within different ADM types, including, but not restricted to, Selective Serotonin Reuptake Inhibitors (SSRIs), Serotonin-Norepinephrine Reuptake Inhibitors (SNRIs), and Tricyclic Antidepressants (TCas). Since antidepressants can be used to treat a number of conditions, the inclusion criteria did not restrict the use of ADM for depression. Rather, it was considered helpful to explore whether the underlying condition influenced decisions on discontinuation / deprescribing.

For the purposes of this review, discontinuation / deprescribing was defined broadly as any process of coming off ADM, whether temporary or permanent, as primary studies on the topic tend to not specify the intended discontinuation duration. This reflects the often uncertain, trial-and-error discontinuation efforts [76].

The type of HP was not restricted – study participants could be GPs, psychiatrists, pharmacists, psychotherapists, nurses or other HPs which may influence or become involved in decisions concerning ADM discontinuation / deprescribing. While GPs play a key role in discontinuation, other HPs also often shape decisions about ADM treatment [77].

The SPIDER search tool (Table 1) was used to identify the main concepts in the research question and structure the inclusion criteria to enable the development of a systematic search strategy [78].

**Table 1:** Organising the inclusion criteria using the SPIDER tool

SPIDER tool	Inclusion criteria
**S** (Sample)	**HPs** *Inclusion criteria*: Any type of HP who may influence and / or become involved in decisions about ADM discontinuation / deprescribing
**PI** (Phenomenon of Interest)	**ADM** *Inclusion criteria*: Any type of ADM, including the most common such as, SSRIs and SNRIs
**D** (Design)	**Qualitative data** *Inclusion criteria*: Qualitative data in the form of, *inter alia*, perspectives, views and beliefs of participants
**E** (Evaluation)	**ADM discontinuation / deprescribing** *Inclusion criteria*: Primary studies which evaluate the perspectives of HPs on ADM discontinuation / deprescribing
**R** (Research type)	**Qualitative collection and analysis** *Inclusion criteria*: Primary studies using qualitative data collection (such as, focus groups and interviews) and analysis, including mixed-method primary studies with a qualitative aspect that can be separately extracted, published in English language

Source: Original work by authors using the SPIDER search tool [78]

### Data sources

We harvested studies from an existing review [75] which conducted systematic database searches from inception to July 2018. Selected key electronic databases representing relevant disciplines including medicine (PubMed via NCBI), nursing (CINAHL via EBSCO*host*) and psychology (PsycINFO via EBSCO*host*) were searched from July 2018 until 1 May 2022. Searches for grey literature were undertaken via Google Scholar, the Networked Digital Library of Theses and Dissertations (NDLTD) and the British Journal of General Practice (BJGP), on 5 May 2022. Searching in Google Scholar stopped after two pages of irrelevant results. Related article checking was performed for the papers which met all the inclusion criteria using the ‘similar articles’ feature in PubMed, and stopped after 100 suggestions. Stopping rules and cut-off values have been used in previous SRs as adequate for ensuring saturation of results, and are in line with recommendations [79, 80]. The PubMed search was repeated on 12 January 2024 to check for studies published since the synthesis was completed.

### Search strategy

A comprehensive search strategy was adopted to seek all available studies. The PubMed search strategy was based on the five main concepts in the research question: HPs, ADM, perspectives / views, ADM discontinuation / deprescribing, and qualitative research. The search terms were developed through iterative pilot searching in order to test and adjust them accordingly.

‘Free-text’ terms were supplemented with controlled vocabulary terms (MeSH terms) where it was demonstrated that they retrieved additional results. The final search query developed for PubMed was adapted for CINAHL, PsycInfo and NDLTD (see Appendix A, Additional File 1). The BJGP and Google Scholar were searched using simplified search queries (see Appendix B, Additional File 1). A search filter for English language was applied where available.

### Screening process

Search results were uploaded into EPPI-Reviewer Web software, a specialised tool for managing information in SRs [81, 82]. Search results from Google Scholar, the NDLTD and the BJGP were screened at source and those considered relevant were imported manually into EPPI-Reviewer for further assessment.

Pilot screening of 50 studies was conducted independently by two researchers (LC and KS) – an inter-rater agreement of 94% was achieved. The titles and abstracts of the remaining studies were screened by LC. For those appearing to meet all criteria, full papers were obtained and screened by both LC and KS independently. Any disagreements were resolved through discussion.

### Data management and extraction

Data extraction was performed in EPPI-Reviewer by LC and checked by KS. Data extracted included bibliographic data, such as, title, author, journal publication and database source, and substantive data including study objective and setting, participant demographics, data collection and analysis method, and summary of findings. Where studies included both HPs and patients as participants, patient information / views would only be extracted through the lens of HPs’ perspectives.

### Synthesis method

Primary study findings were synthesised using thematic synthesis [83] and were further analysed using the Social Ecological Model (SEM) [84] and a logic model.

For the thematic synthesis, once multiple readings of the studies were conducted to achieve immersion, LC performed inductive ‘line-by-line coding’ of the findings, including both participant quotes and authors’ interpretations, to identify common and emergent themes. Higher level ‘descriptive themes’ and interpretive ‘analytical themes’ were developed through discussion between LC and KS in an iterative manner.

The dynamic interrelationship of the themes was further explored using Bronfenbrenner’s (1977) SEM and a logic model. The SEM, is commonly used for demonstrating the contextual environments surrounding policy issues across different societal dimensions [85], while logic models are considered valuable for understanding complexity, supporting conceptual thinking, and illustrating hypothesised causal links [86, 87]. The SEM was used to locate themes related to ADM discontinuation within relevant systemic levels. Subsequently, the logic model was applied to explore how and why these themes may influence one another within and across levels, helping to identify the most impactful factors for targeted policy interventions.

### Quality appraisal and sensitivity analysis

Using criteria developed by the EPPI-Centre [88, 89] and abiding by principles of good practice for conducting social research with the public [90], the quality of each study was considered according to: rigour of sampling; data collection and analysis; whether study findings were grounded in / supported by data; whether the breadth and depth of findings were adequate for the review; and, whether participants’ experiences / perspectives were privileged. Ratings from these assessments were used to consider each study’s Reliability and Relevance, which were then combined using a predefined algorithm to determine overall Usefulness: studies rated High on both were classified as ‘Gold Standard’, those with one High and one Medium as ‘High’, both Medium as ‘Medium’, and any Low rating as ‘Low’. The full QA tool can be found in Appendix C, Additional File 1, while the algorithm for the overall Usefulness rating can be found in Appendix D, Additional File 1.

The overall Usefulness rating informed the interpretation of a sensitivity analysis exploring which studies contributed to the development of each descriptive theme. Studies were not excluded based on their assessed quality, rather, it was expected that weaker studies would contribute less to the development of themes and overall synthesis findings [91]. QA and sensitivity analysis were conducted by LC and checked by KS, with any disagreements resolved through discussion.

### Reporting checklists

This review was guided by the Preferred Reporting Items for Systematic reviews and Meta-Analyses (PRISMA) checklist and the PRISMA checklist for review abstracts [92]. Items which were not relevant to this review were indicated as ‘N/A’. Both PRISMA checklists can be found in Appendix E, Additional File 1. The ENTREQ statement was also consulted to supplement reporting checks more suited for certain stages of qualitative evidence syntheses (QES) [93]. This is found in Appendix F, Additional File 1.

## Results

### Screening results

Upon removal of duplicate studies (*n* = 80), identified studies were initially screened on title and abstract resulting in the exclusion of 838 out of the total 862. Twenty-four studies were full-text screened; ten were excluded resulting in 14 studies for synthesis.

**Fig. 1:**
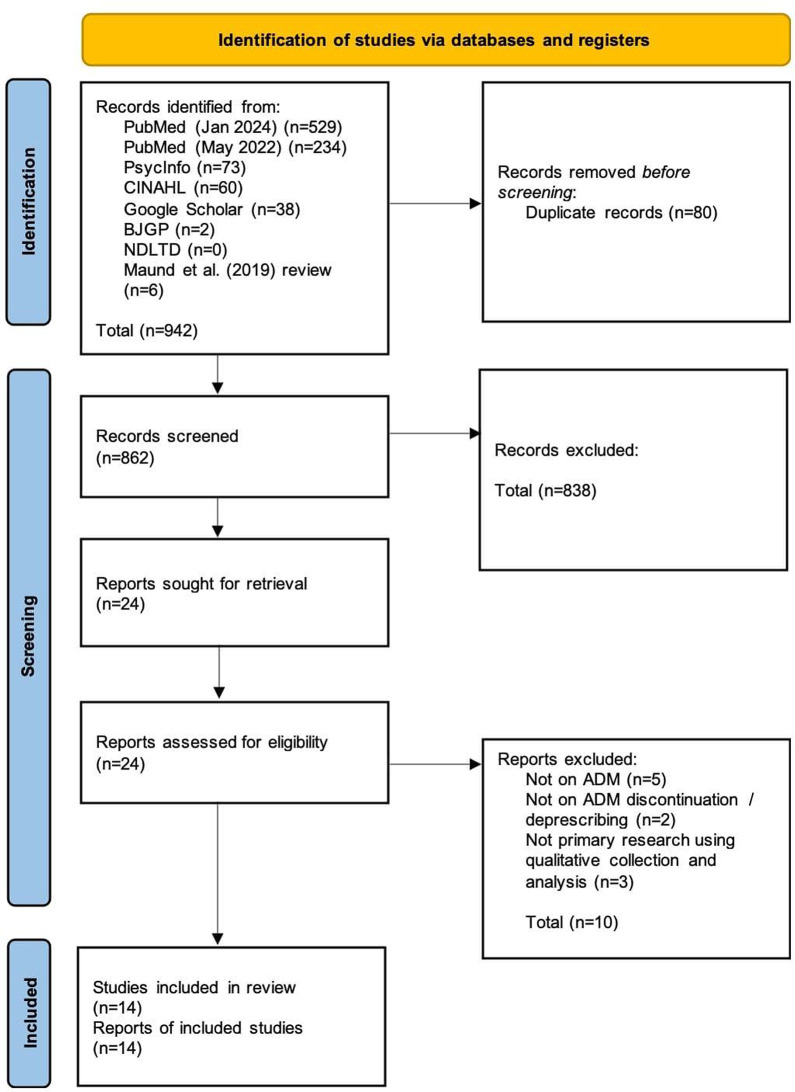
PRISMA flow diagram applied to this review. Source: Page et al., 2021 [92]

The PRISMA flow diagram (Fig. 1) illustrates the studies identified and the decisions taken at each stage of the screening process.

### Overview of included studies

Included studies were published between 2002 and 2022, and were conducted in Denmark [94], Belgium [95, 96], Australia [97], Ireland [98], Norway [99], England [77, 100–102], Scotland [103], the US [104] and the Netherlands [105, 106].

Study participants included: GPs only [95–98, 101, 103, 105]; GPs and nurses [99, 100], psychiatrists only [104]; GPs, GP assistants, nurses, health team workers and psychotherapists [77]; GPs, nurses, psychiatrists and psychologists [106]; GPs, a pharmacist and nursing home staff [94]; and GPs, practice counsellors and pharmacists [102]. Four studies also included patients as participants [101, 102, 105, 106], and one included patients and patient relatives [94].

Three studies were undertaken in nursing home settings [94, 95, 99]. The rest described their setting as primary care [96–98, 100–103, 105], primary and secondary care [77, 106], or was unspecified [104].

Eight studies described their analysis method as thematic analysis [77, 94–98, 100, 102], while two appear to have employed similar methods [99, 105]. Other methods included discourse analysis [104], framework analysis [101, 103] and concept-mapping [106].

More information on study characteristics and summary of findings can be found in Appendix G and Appendix H, in Additional File 1, respectively.

### Quality and relevance of included studies

QA results of the included studies are presented in Table 2. Most were assessed as having either a Gold standard or a High rating of overall Usefulness.

**Table 2:**
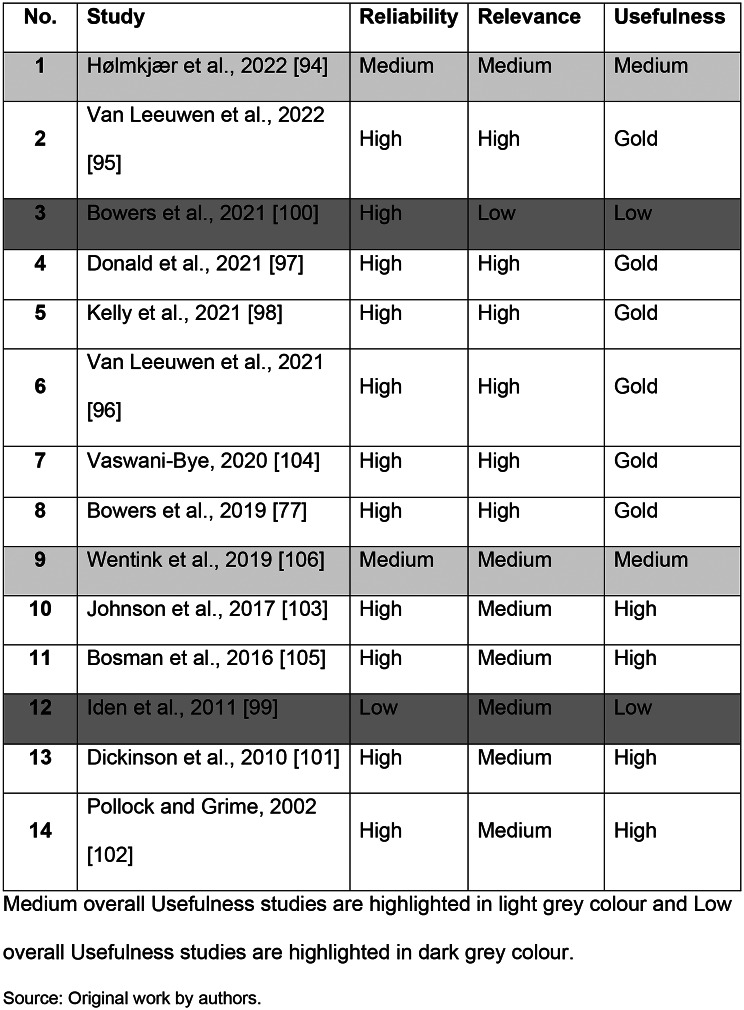
Reliability, relevance and overall usefulness ratings of included studies

Seven studies were found to have Medium Relevance to the current review [94, 99, 101–103, 105, 106] since none of their aims were exclusively concerning ADM discontinuation / deprescribing. Two studies were rated as having Medium Reliability – one on the basis of having limited depth [106] and the other due to weak sampling, and data collection and analysis methodology and reporting [94]. Two studies were rated as having Low Usefulness, since one had a Low Relevance rating [100] and one a Low Reliability rating [99]. The Low Relevance study focused on HPs’ views on a digital tool supporting ADM deprescribing instead of their wider perceptions on barriers and facilitators on ADM discontinuation / deprescribing. The Low Reliability study was viewed as lacking transparency in its methods and sampling, and having limited depth.

### Synthesis findings: analytical and descriptive themes

The thematic synthesis yielded eight overarching analytical themes and 50 descriptive sub-themes. Descriptive sub-themes are divided into barriers and facilitators; analytical themes present a more abstract level of analysis and can thus be understood as both barriers and facilitators. The full list of descriptive sub-themes can be found in Appendix I, Additional File 1.

**Fig. 2:**
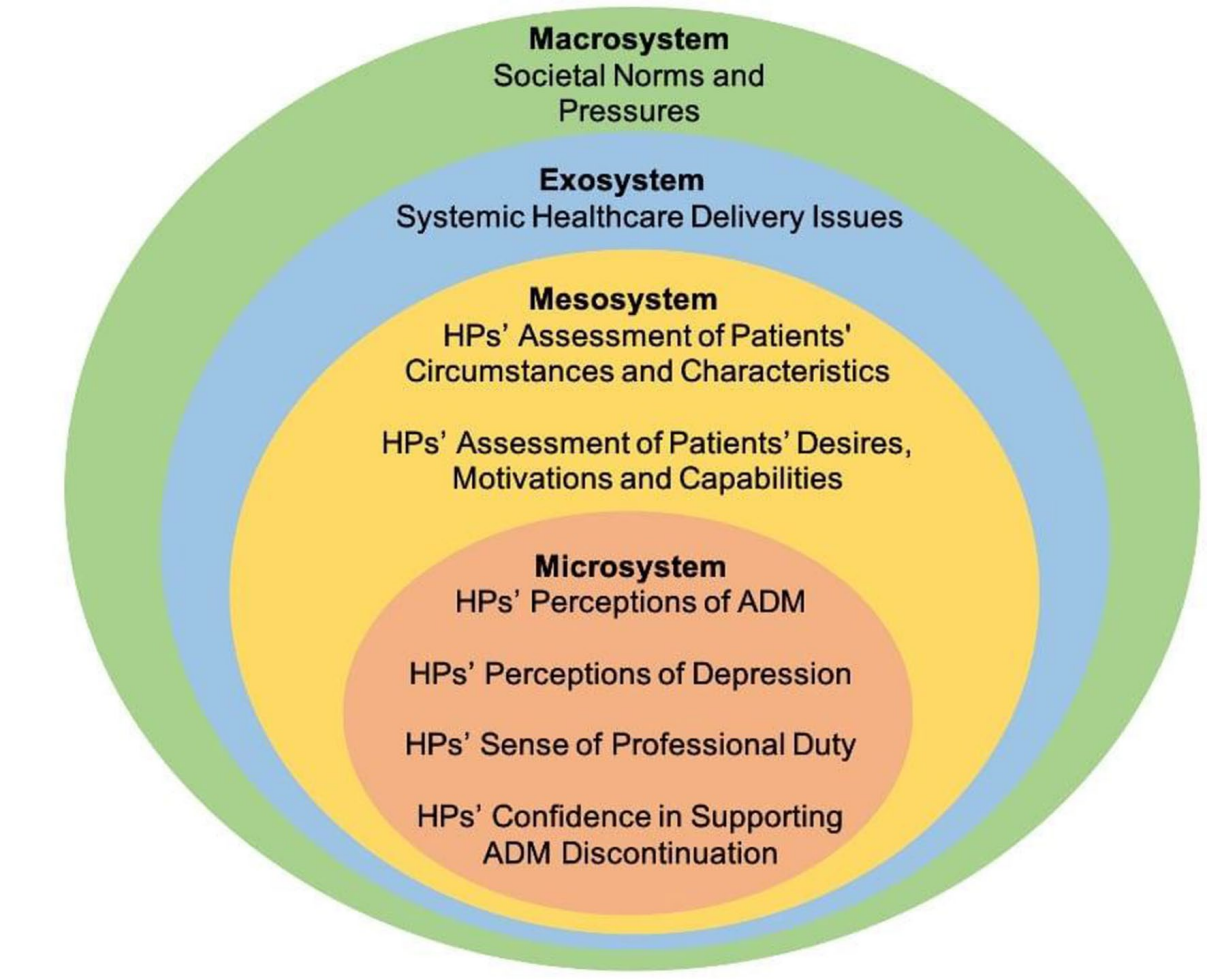
Analytical themes of ADM discontinuation from HPs’ perspectives mapped against the SEM. Source: Authors’ own work adapted from Bronfenbrenner (1977) [84]

The analytical themes were further analysed using Bronfenbrenner’s (1977) SEM. Figure 2. presents the overarching analytical themes mapped against the SEM’s microsystem, mesosystem, exosystem and macrosystem.

Below, analytical and descriptive sub-themes are discussed under their corresponding SEM societal dimension. Quotes from study participants or authors are reported to exemplify key points.

#### Microsystem

The microsystem in the SEM considers policy issues as nested within the individual and encompasses direct experiences and relationships that influence personal attitudes, beliefs, values, knowledge and capabilities [107]. The first four analytical themes were found to fit under this dimension since they concern HPs’ personal perceptions of ADM and depression, their sense of professional duty and their confidence in supporting ADM discontinuation.

##### HPs’ perceptions of ADM

The way HPs characterise ADM and perceive its mechanism of action appears to shape their views on whether it constitutes an ideal treatment for depression and on discontinuation / deprescribing.

Some HPs characterised ADM as safe and effective, due to their perception that side-effects are minimal and mild, and that ADM treats depression either by fixing a chemical imbalance in the brain or through the placebo effect [95, 96, 101–104]:

*[GP:] I usually use – “Look you know if you’ve got a broken leg then you have a crutch and so as far as I’m concerned if you’ve got a broken brain then you’ll need a crutch there as well and this is what these chemicals are”.* [102], p. 29.

Some HPs felt that ADM should be prescribed as the primary treatment for depression since it was considered more valuable than ADM alternatives, due to their perceived ability to quickly yield positive results [95, 97, 101–104]. Given that ADM was considered safe and effective, some believed that deprescribing is a negative intervention, with no benefits [95, 96]:

*[GP:] When you start up medication*,* you expect a positive result and people feel better*,* but with quitting nothing ‘happy’ is going to come from it*,* … stopping will not immediately affect the patient.* [96], p. 538.

On the other hand, other HPs were cautious of ADM, since they perceived them as being unnatural, addictive / toxic, and doubted their overall efficacy [96, 102, 104, 105]. They believed that other alternative approaches should be preferred, especially for mild / moderate depression [103, 104]:

*[Psychiatrist:] I think [therapy is] way more useful and way more long-lasting*,* way more empowering.* [104], p. 47.

Some HPs supported discontinuation since they believed that it would enable patients gain back their sense of personal agency, invest in psychological change, and alleviate suffering from side-effects [97, 104]:

*[GP:] The patient gains new life*,* they feel like*,* ‘I don’t need it! I’ve regained my life. I don’t need to be dependent on anything’.* [97], p. 511.

##### HPs’ perceptions of depression

HPs’understandings of what causes depression can influence their views around ADM treatment and discontinuation / deprescribing.

On the one hand, some HPs believed that depression is a chronic, medical illness [95, 99, 101, 102, 104, 105] and was often compared to conditions like diabetes. Based on this view, continued ADM use was deemed necessary:

*[Psychiatrist:] Well*,* there’s something wrong with their serotonin […] taking the medication prevents depression and treats it* [104], p.40.

On the other hand, some HPs viewed depression as an understandable reaction to challenging life circumstances [97, 99, 101, 102, 104]. Consequently, ADM was believed to merely help alleviate depression symptoms instead of treating the underlying cause:

*[GP:] […] if you accept that the vast majority of people have a reactive depression […] they [antidepressants] improve mood but unless you*,* unless you deal with the cause of the depression it is going to return.* [102], p. 28.

##### HPs’ sense of professional duty

HPs’ differing views on their duty in relation to patients suffering from depression or taking ADM treatment seem to influence whether they prioritise deprescribing.

HPs who perceived ADM as safe and effective, considered it their duty to alleviate patients’ suffering through the medication and noted that withholding it from them was unethical [95–97, 101, 103, 104]. They also believed that ADM validated the illness and gave hope to patients that there is a treatment for it [101, 103, 104]:

*[GP:] There are problems that we all have in our life. Some people need to have it turned into a medical problem to make it more valid or something.* [101], p. 148.

Some HPs did not perceive it their duty to initiate discussions around discontinuation with their patients and believed that these conversations should be broached by patients themselves [77, 95, 96, 98, 100, 105]. HPs were also reluctant to question other HPs’ or patients’ decision to continue ADM [94–98, 101, 102, 104].

Moreover, HPs often viewed deprescribing for stable patients as an unnecessary intervention and considered maintenance treatment satisfactory [77, 94, 95, 97, 99, 100, 105].

In contrast, other HPs believed it was their duty to improve their patients’ situation and protect them from adverse ADM effects by encouraging discontinuation [95, 97]. A small number of HPs explicitly stated that the responsibility of initiating discontinuation lies with the prescriber [77].

To achieve successful discontinuation, many HPs believed they should invest in the therapeutic relationship of consultations [95, 97, 98, 102–104, 106], and focus on patients’ strengths and sense of agency [97, 100, 106].

##### HPs’ confidence in supporting ADM discontinuation

Several factors were found to jeopardise or enhance HPs’ confidence in supporting ADM discontinuation.

Fears and insecurities of HPs were often a reason for avoiding ADM deprescribing [77, 94–99, 101, 103–106]. HPs feared that by attempting deprescribing they would jeopardise their relationship with patients, patients’ relatives, or other HPs who resisted such decision. Most importantly, they feared they would destabilise the situation of patients and be held accountable for their relapse:

*[GP:] […] you may have made the wrong decision and now caused a medical problem for somebody who was well*,* through interfering with their medications.* [98], p. 6.

Other HPs noted that their experience in pharmacological treatments made them idiosyncratic or paternalistic on depression management leading them to encourage ADM use [103, 104]:

*[Psychiatrist:] I try to be very patient-centered about this [prescribing] but I end up being paternalistic without meaning to be. Because I know more about these medicines and they’re coming to me for them.* [104], p. 43.

Conversely, being transparent about the medical uncertainty and ADM risks was found to be a facilitator to discontinuation [94, 97, 102, 104, 106]. Some HPs considered the possibility of relapsing as not prohibitive for attempting discontinuation since they accepted deprescribing as a ‘trial-and-error’ journey [102, 104, 106]:

*[GP:] This is why when you actually stop treatment you have to make it clear to them that you are just testing the water.* [102], 50.

#### Mesosystem

The mesosystem refers to interpersonal relationships in which a person’s individual microsystems do not function independently, but instead interact with each other and assert influence upon one another [107]. Whereas this is often used to encompass relationships with family and friends, in the context of HPs, it can be understood as encompassing their relationships and interactions with patients. The analytical themes under this societal dimension concern patients’ circumstances, characteristics, desires, motivations and capabilities and the way HPs assess and respond to these in relation to ADM discontinuation / deprescribing.

##### HPs’ assessment of patients’ circumstances and characteristics

According to HPs, patient circumstances and characteristics may constitute barriers or facilitators to ADM discontinuation / deprescribing.

HPs considered old age or living in a nursing home to be a barrier to discontinuation due to their belief that older people are psychologically fragile and that living in a nursing home is depression-enhancing or challenging to adjust to [94, 96–99, 101].

Drug addiction or polypharmacy were also viewed as a barrier to discontinuation given that HPs prioritised helping patients rehabilitate or reduce other, more risky medication [96, 98, 104, 106]. HPs additionally believed that discontinuation is unlikely to succeed if patients had been taking ADM for many years, or if they had previously unsuccessfully attempted discontinuation [97, 98, 101–104, 106]. Family history of depression [104, 106] or severe / recurring depression [98, 101, 103, 104, 106] were also thought to hinder discontinuation.

Other patient circumstances were thought to favour discontinuation, such as, strong support network (e.g. family, relatives, friends) [77, 98, 106], mental, financial and relationship stability, and absence of current or upcoming stressful life events [97, 98, 102, 106]. A few HPs stressed that engagement in self-care and healthy lifestyle were important for successful discontinuation [97, 98]. Positive life events were also considered a facilitator since they were thought to distract patients from withdrawal symptoms and ‘replace’ the lost positive effect of the medication [95, 96]:

*[GP:] When you think that removal of the tablet is not going to make a difference because they are busy with all sorts of things*,* e.g. they are happy because of a grandchild*,* you should do it [deprescribe].* [96], p. 536.

Additionally, HPs encouraged discontinuation in circumstances where patients experienced certain health issues (such as severe ADM side-effects or pregnancy) which could be further compromised because of ADM use [95, 96, 105, 106]. Dementia was also thought to facilitate deprescribing since it was considered unlikely that patients would resist discontinuation (due to limited comprehension) and because of perceived ADM ineffectiveness in these patients [94, 95]. Lastly, HPs believed that in contrast to severe / recurring depression, mild depression constituted a facilitator to discontinuation [98, 103, 104].

##### HPs’ assessment of patients’ desires, motivations and capabilities

Studies indicated that HPs often assess ‘readiness’ for ADM discontinuation based on patient desires, motivations and capabilities. These are either explicitly expressed by patients themselves, or inferred by HPs, based on their own understanding of a patient’s situation.

HPs often assumed that patients who did not broach the subject of ADM discontinuation wanted to stay on the medication [77, 95, 100] or they generally believed that patients were psychologically dependent on ADM and therefore needed to continue treatment [97, 101–104]:

*[Study authors:] GPs repeatedly likened the process of antidepressant discontinuation to smoking cessation or dependence management […]* [97], p. 511.

HPs also thought that patients sometimes lacked the motivation or ability to follow ADM alternatives, such as psychotherapy, which hindered discontinuation [95, 96, 101]. In the case of older people, HPs assumed that they were disinterested in their medication treatments and therefore considered it unimportant to discuss discontinuation with them [95].

Conversely, when patients actively requested to discontinue ADM [95, 97, 101, 102, 104, 105] or seemed to be aware of their depression / anxiety triggers [97], HPs felt that discontinuation was more possible.

#### Exosystem

SEM’s exosystem refers to organisational aspects of a policy issue including formal and informal social structures and institutions such as “the world of work” and “agencies of government” which may not necessarily contain the individuals but influence them indirectly [84], p. 515. Thus, in relation to this review, systemic healthcare delivery aspects around ADM discontinuation / deprescribing are associated with this societal dimension.

##### Systemic healthcare delivery issues

HPs stressed how certain systemic healthcare delivery issues may constitute barriers to ADM deprescribing and how they use their experiential learning, support network and collaborative decision-making to tackle these.

One of the most prevalent sub-themes was the unavailability / inaccessibility of HPs due to their busy schedules, short consultation time with patients or in certain circumstances, lack of private space to discuss ADM issues with patients [77, 94–98, 100–103, 105]:

*[GP:] Sometimes you are just very busy and you think “let’s get those repeat prescriptions over and done with”.* [105], p. 712.

Moreover, cases where patients’ treatment was initiated by another HP or there was split care between HPs were considered a barrier to discontinuation due to lack of continuity and inadequate familiarity with patients [77, 94, 96, 98, 104, 105].

Another barrier to discontinuation was the inaccessibility and unavailability of ADM alternatives such as psychotherapy [94–99, 101]:

*[GP:] There are no cognitive services freely available […] I’m not going to get priority if I say I want to stop a SSRI and I want you to provide cognitive support.* [98], p. 5.

Many HPs also stressed the inaccessible, unclear or insufficient evidence on diagnosing depression, long-term ADM effects, or ADM deprescribing [77, 95–102, 104, 105]:

*[Nurse:] We are not skilled in differentiating between these conditions. If they cry*,* we call it depression and give them antidepressants. And that’s it.* [99], p. 253.

*[GP:] I think that long-term evidence is actually quite limited about any harms of long-term use. It is very difficult because most of the trials with antidepressants are short-term.* [97], p. 513.

HPs noted the lack of relevant tools (e.g. prescription systems) and training (e.g. enhancing HPs’ communication skills) for supporting deprescribing [77, 94–100, 105]:

*[GP:] Repeat prescription systems*,* maybe a notice when you’re a printing them off*,* not even specific to SSRIs but some sort of a notice like have you taken the opportunity to deprescribe at this time.* [98], p. 5.

Some HPs described a professional hierarchy within the medical system and highlighted poor communication among them, which deterred effective collaboration in decision-making around ADM deprescribing [94–96, 98, 102, 104]. Notably, this issue was raised by nurses or pharmacists with no authority to prescribe medication or GPs who felt they did not have the specialised knowledge of psychiatrists:

*[Study authors:] […] some pharmacists were deferential towards the prescriber*,* assuming superiority of the prescriber’s knowledge.* [102], p. 57.

One way HPs overcame systemic barriers was by developing their own techniques around deprescribing, such as, development of tapering strategies based on factors like ADM type, treatment duration, time of year (e.g. avoiding discontinuation during winter) and patient response [95, 97, 98, 101–106]:

*[Psychiatrist:] I made it [tapers] up and then I altered it*,* I think*,* based on how people did with that…I’ve never seen any guidelines.* [104], p. 65.

HPs engaged in collaborative decision-making with other HPs, patients’ relatives or patients, as a way of splitting responsibility around deprescribing. This involved sharing knowledge and tips around deprescribing and ADM in general [77, 94–98, 101, 102, 104–106]. However, mandatory involvement of relatives in decision-making was viewed as potentially problematic due to time-limitations and the over-protectiveness of relatives for their loved ones which could discourage deprescribing efforts [94].

Lastly, HPs noted that setting treatment duration and goals with their patients at ADM initiation helped them monitor the treatment process and consider deprescribing early on [77, 95, 97, 101, 104, 106].

#### Macrosystem

The macrosystem refers to a more abstract societal level rather than specific environmental contexts and is described as the “general prototypes, existing in the culture or subculture that set the pattern for the structures and activities occurring at the concrete level” [84], p. 515. Such cultural ‘blueprints’ are carried in the minds of society members as ideologies and manifest through customs and patterns in everyday life. ADM societal norms and pressures are thus presented under this dimension and highlight shared beliefs in relation to medicalisation of health that can shape HPs’ practice priorities and actions.

##### Societal norms and pressures

The potential impact of societal norms on HPs’ deprescribing practices is illustrated by an apparent wider tendency towards overreliance on ADM and the encouragement of long-term prescribing.

HPs observed that pharmaceutical companies focused on developing new drug treatments and disincentivised efforts on deprescribing, including providing insufficient funding on discontinuation / deprescribing research [97, 103, 104]:

*[Psychiatrist:] I believe that it [deprescribing] is a hard question to study and that there is less vested interest in our medical economy in spending money on this question versus the question of new tools*,* new agents*,* different things to try.* [104], p. 62.

Some HPs operated on the underlying rule within the medical community that once ADM treatment is initiated, deprescribing is not prioritised. This often resulted in a blind acceptance of repeat prescribing as an established standard of care [77, 95–97, 104, 105]:

*[GP:] There is a little bit of an underlying rule that once you start this medication nobody thinks to stop it.* [97], p. 513.

HPs noted that patients, relatives or other HPs often had an expectation that depression or sorrow needed to be treated with ADM. This put pressure on HPs to prescribe or continue ADM [94–96, 99, 101, 103, 104]:

*[GP:] They [patients] feel that unless they are on a tablet for it then they are not having any treatment.* [101], p. 149.

The only facilitator to deprescribing found under this theme was HPs’ awareness of these influences which helped them to actively resist overreliance on medication [97, 101, 103, 104]:

*[GP:] …there was the Defeat Depression campaign […] pharma were probably being very very clever there*,* and more subtle than usual. I would say…people get quite well develop antibodies to pharma now. So they actually probably have to work harder to convince me.* [103], p. 7.

### Sensitivity analysis results

The sensitivity analysis (see Appendix J, Additional File 1) identified that two studies rated with Low Usefulness [99, 100] contributed only to 6 and 11 themes respectively, while two Medium Usefulness studies [94, 106] contributed only to 13 and 14 themes respectively. None of these studies contributed an original theme to the overall synthesis so excluding them would only reduce fine detail rather than change the final synthesis outcomes. The four most common themes were HPs’ fears, alongside systemic issues related to HP unavailability, insufficient knowledge on depression and ADM, and the need for support and collaborative decision-making.

### Logic model

**Fig. 3:**
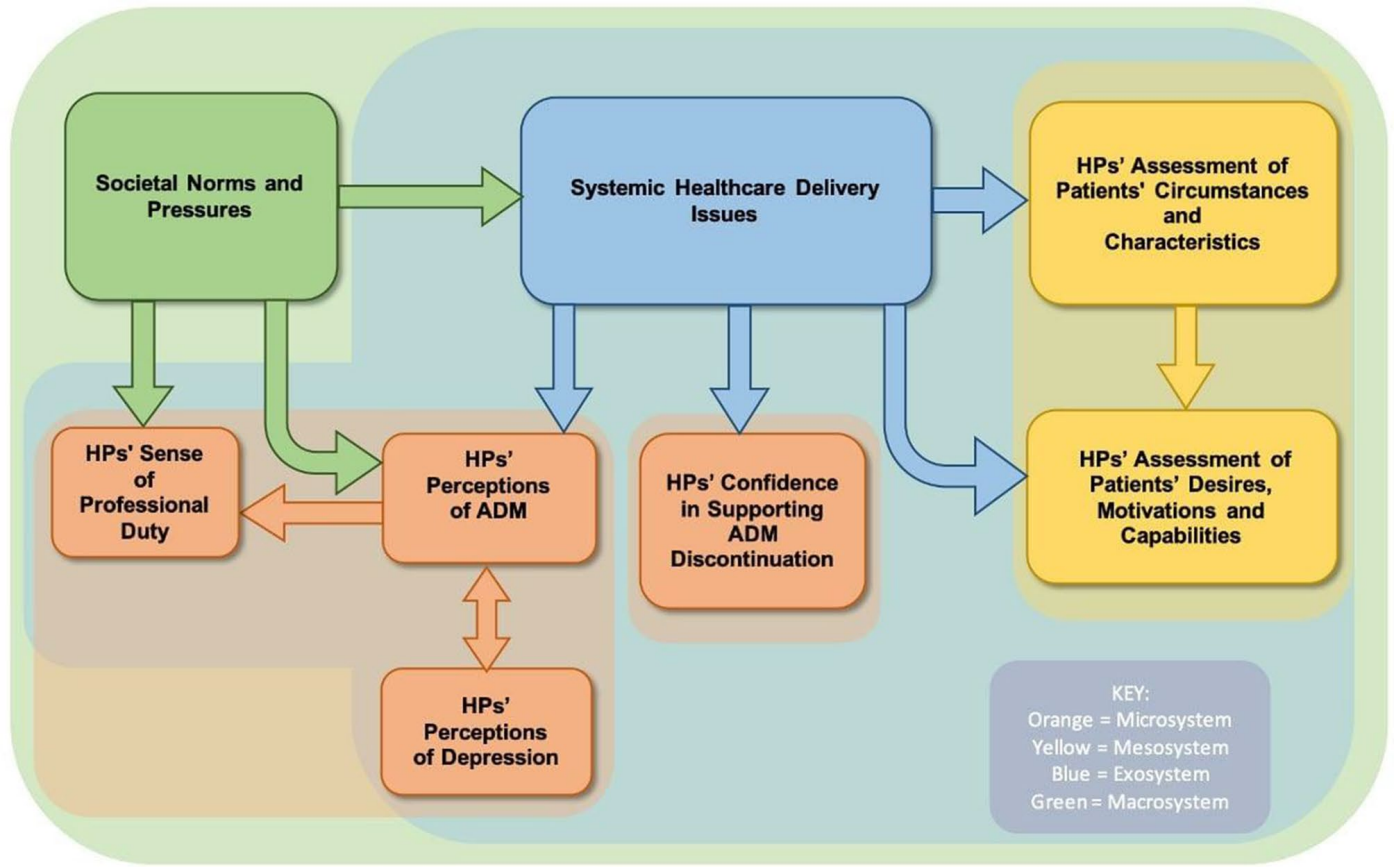
A logic model of the analytical themes. Source: Original source by authors

Successful policy-making ideally involves addressing issues at all levels of the SEM, yet, this model appears to lack guidance on issue prioritisation. A logic model, shown in Fig. 3, was used to detail interconnections between themes and reveal how and why these connections may operate. The arrows illustrate direct relationships between the themes, whereas the background colours illustrate the sphere of influence of each overarching theme towards the overall systemic structure (microsystem in orange, mesosystem in yellow, exosystem in blue, and macrosystem in green).

The model shows that ADM and depression perceptions may be linked, potentially impacting HPs’ professional duty (microsystem). Perceiving depression as a medical illness aligns with the belief in ADM’s efficacy in treating brain chemical imbalances. HPs viewing ADM as safe and effective in turn may feel obligated to prescribe it for patient relief.

It is also demonstrated that HPs often evaluate patients’ desires, motivations and capabilities based on their patients’ personal circumstances and characteristics (mesosystem). For instance, HPs might avoid deprescribing for older patients due to perceived life stability risks at old age and assumptions about psychological dependency.

Systemic healthcare delivery issues (exosystem) appear to influence all themes and SEM levels, except wider societal factors. Lack of evidence on ADM may be affecting HPs’ perceptions, steering them toward established beliefs favouring medication (microsystem). Absence of deprescribing guidelines, training or tools, and lack of ADM alternatives can be influencing HPs’ confidence and exacerbate their fears in relation to deprescribing (microsystem). Additionally, it potentially heightens reliance on patient circumstances and support networks, and hinders effective HP-patient communication about patients’ true desires, motivations and capabilities (mesosystem).

Lastly, societal norms and pressures (macrosystem) appear to impact all themes and SEM levels, echoing Bronfenbrenner’s characterisation of the macrosystem as the ‘blueprint’ of society manifesting itself through the other levels. Medication over-dependence may be reducing interest in studying and training HPs on deprescribing and in promoting alternative treatments leading to systemic healthcare delivery issues (exosystem). The shift towards biological psychiatry seems to directly shape ADM perceptions as the primary treatment for depression which in turn can impact how HPs perceive their role, often feeling pressured to validate depression through medication (microsystem). Moreover, the culture of ‘repeat prescribing’ within the medical field can be directly influencing HPs’ sense of professional duty because of the established reluctance to prioritise deprescribing and initiate discussions on discontinuation (microsystem).

Overall, the logic model suggests that none of the themes exist in isolation but are rather interconnected both within and across societal levels. The two overarching analytical themes relating to societal norms and pressures, and to systemic healthcare delivery issues, are found to have a higher significance due to their wider apparent impact.

## Discussion

### Principal findings

A thematic synthesis of 14 studies reporting HPs’ perspectives on the barriers and facilitators to ADM discontinuation / deprescribing yielded eight overarching analytical themes and 50 descriptive sub-themes, which were further analysed using the SEM and a logic model. Systemic healthcare delivery issues, and societal norms and pressures, emerge as likely the most impactful barriers given their observed far-reaching influence on all other aspects on ADM discontinuation, such as, HPs’ perceptions of ADM and depression, their sense of professional duty, their confidence in supporting ADM discontinuation and the way they assess their patients’ circumstances, characteristics, desires, motivations and capabilities. For addressing the most prominent issues, HPs reported engaging in collaborative decision-making, creating personalised tapering techniques, planning ADM treatment duration and goals at treatment initiation, and being mindful of a general overreliance on medication.

### Strengths and limitations

This review contributed to filling the evidence gap in relation to HPs’ perspectives on the barriers and facilitators to ADM discontinuation, which is acknowledged as an important policy issue. A rigorous and transparent search was undertaken using a variety of data sources and new studies have successfully been identified, most of which were rated as having a Gold overall Usefulness. Fourteen studies were included which exceed a suggested threshold of 12 for carrying out a robust synthesis [108]. One study [94] was identified through the repeated PubMed search in January 2024 and was found to be largely confirmatory of the existing findings. Although the search was not updated beyond January 2024, a scoping search on Google Scholar revealed two recent primary studies that broadly corroborate our findings, indicating limited conceptual developments in the field [109, 110]. The review included studies undertaken in different countries and captured perspectives of a variety of HPs, including, GPs, psychiatrists, psychologists, nurses and pharmacists.

The sensitivity analysis maximised transparency. Although some themes were supported by a small number of studies, this means that some studies provided original input to the synthesis. Use of theory and logic models within SRs and QES is increasingly being recognised as important [111]. Analysis using the SEM and a logic model enabled deeper and more insightful conclusions which went ‘beyond’ the data of the original studies and pointed towards factors likely to have the greatest impact on ADM discontinuation.

There are some notable limitations however. The review predominantly captures GP perspectives; fewer studies included other types of HPs. Since all included studies were written in English, this review is limited by language bias. Development of themes and sub-themes was performed by a single researcher and further developed through discussion between two researchers. Although not as robust as two reviewers working independently, this method is used in other published QES [112]. Lastly, whereas the review included studies conducted across different countries, we did not identify findings that illuminate how variations in healthcare systems influence effective ADM discontinuation. This represents a potential avenue for future research.

### Comparison to existing research

Through this review we sought to add valuable insight into the existing body of research on ADM discontinuation. In comparing our findings with other literature, we found that our review not only identifies themes consistent with previous research but also offers some original findings and a distinctive interpretation of how these interrelate – allowing us to highlight the factors likely to exert the greatest influence.

Many of the themes identified in our review align with those found in earlier work exploring patients’ perspectives on ADM discontinuation [75]. For instance, both highlight how perceptions of ADM and depression can act as either barriers or facilitators to discontinuation. Similarly, themes in our findings relating to patients’ desires, motivations, capabilities, and personal circumstances and characteristics, closely reflect those identified in the patient-focused review. This alignment suggests that patients’ self-assessments strongly echo how HPs perceive and evaluate readiness for discontinuation. There is also agreement between patient and HP perspectives on systemic issues in healthcare delivery - such as inadequate information about discontinuation and ADMs, lack of regular reviews, time constraints and limited support from other professionals. Both patients and HPs also recognise that HPs can exert authority over discontinuation decisions and thus influence patients’ choices, while there is mutual anticipation about which party will initiate discussions around stopping treatment.

In addition to these shared insights, our review contributes unique perspectives specific to HPs. Notably, certain themes related to professional self-identity - such as a sense of duty and confidence in supporting discontinuation - are either absent or less developed in the patient-focused literature. For example, while the earlier review briefly noted that GPs may fear destabilising patients under a broader theme of ‘Fears’ [75], our findings delve deeper, uncovering concerns about professional consequences. These include anxieties around being held accountable for negative outcomes and fears of damaging relationships with patients, their families, and colleagues. These findings are consistent with literature on ‘defensive medicine’ defined as “a deviation from standard medical practice due to fear of malpractice liability claims” [113], p. 1. Another distinctive theme relates to how HPs view ADM prescribing as a means of validating patients’ negative life experiences, by framing their symptoms as medical issues and offering hope through a trusted treatment. This underscores the symbolic role of ADMs in patient care, which can serve as a rationale against discontinuation. Most importantly, while the patient perspective review identified pressures to continue ADM - particularly from significant others - it did not reflect broader societal forces, such as the growing emphasis on biological psychiatry or the culture of ‘repeat prescribing’.

In relation to other QES on discontinuation of medications not specific to ADM, our review similarly identifies recurring themes related to challenges in healthcare delivery. Two recent QES explored HP perspectives in relation to overall medication or psychotropic drugs among older population [71, 72], while another two focused on the perspectives of HPs, patients, and other stakeholders regarding the discontinuation of benzodiazepines and Z-drugs [73, 74]. These pointed to gaps in training, knowledge and resources, the importance of interprofessional collaboration, and the lack of consultation time. However, our review also suggests that the lack of easily accessible ADM alternatives, such as psychotherapy, may be an important discontinuation barrier specific to ADM.

Notably, our review offers a methodological contribution by demonstrating how the combined use of the SEM and a logic model, as applied in this analytical approach, can reveal possible interconnections and enhance thematic synthesis findings, identifying factors with the greatest apparent impact. Specifically, we show how healthcare delivery issues may influence a range of other factors affecting ADM discontinuation, including HPs’ psychology, perceptions, and behaviours. Our approach also uncovered the pervasive role of societal norms and pressures, which directly or indirectly may shape all aspects of ADM discontinuation. This is echoed by wider literature indicating that pharmaceutical marketing strategies can strongly influence HPs’ new drug uptakes and prescribing behaviours - contributing to issues such as a lack of transparency and accountability, weak enforcement of legislation, and even corruption, particularly in low- and middle-income countries [114–118]. In line with these observations, our findings suggest that a growing emphasis on biological psychiatry and a culture of ‘repeat prescribing’ may be key underlying drivers of both systemic healthcare delivery challenges and the psychological, perceptual, and behavioural barriers that impede ADM discontinuation.

### Implications for research and practice

While the evidence suggests that a multi-layered approach would be ideal for maximising policy effectiveness, priority could be given to interventions addressing the two themes demonstrating the greatest impact, as identified by the logic model: societal norms and pressures, and systemic healthcare delivery issues.

However, few deprescribing interventions appear to target the barriers identified in the exosystem and macrosystem levels [10, 119]. Similarly, whilst UK guidelines for the treatment of depression - including ADM deprescribing - have been updated to acknowledge the potential severity of side effects and withdrawal symptoms [33, 120], they primarily address practical and procedural barriers to deprescribing (e.g. tapering techniques, shared decision-making and continuity of care). In the same vein, the 2024 Maudsley Deprescribing Guidelines, while filling certain knowledge gaps, do not fully tackle organisational and behavioural issues [119, 121].

General deprescribing principles, not specific to ADM, offer useful frameworks into which ADM discontinuation interventions can be integrated [122, 123]. These frameworks promote a cyclical-step approach: beginning with awareness and education, reviewing current medications to identify those that can be discontinued, planning a deprescribing strategy in partnership with patients and carers, providing ongoing support and review, and offering feedback to staff about deprescribing practices [122].

Workshops or seminars for HPs that explore issues such as the medicalisation of health and the pharmaceutical industry’s influence on prescribing behaviours, along with information campaigns directed at medical institutions and health centres, could help shift established societal norms and normalise the use of ADM alternatives. Education and training are already implemented as interventions within the general polypharmacy deprescribing efforts [124] and align with the first deprescribing principle - raising awareness - of the abovementioned framework [122].

To tackle systemic healthcare delivery issues further, emphasis could be placed on addressing evidence gaps so that HPs can access robust evidence on ADM and depression. This includes evidence on long-term effectiveness and side-effects of ADM, along with comparative long-term benefits of alternative treatments [10, 54, 125–128], the effectiveness of tapering techniques - across different ADM types, treatment durations, population groups, and underlying condition - [76, 129–131], on improving validity in differentiating between withdrawal symptoms and depressive relapse [132], and evidence related to the ‘medical illness’ model of depression [54]. Additional systemic interventions could focus on strengthening collaborative decision-making and improving access to ADM alternatives. Tools, such as automated alerts for repeat prescriptions could also support HPs in managing deprescribing processes more efficiently. These actions are again consistent with deprescribing principles - such as raising awareness / educating HPs, fostering partnership among HPs, patients and carers, and conducting ongoing reviews [122] - and also align with existing interventions, e.g. electronic medical control alerts, in related fields [124, 133, 134].

## Conclusion

Through use of the SEM to structure our analysis, and by development of a logic model, we identified that barriers and facilitators to ADM discontinuation / deprescribing are interconnected both within and across societal structures, and we uncovered reasons and mechanisms by which these connections may operate. Societal norms and pressures, and systemic healthcare delivery issues, appear to directly or indirectly influence all aspects around ADM discontinuation / deprescribing. As such, further research and interventions targeting factors at these broader societal levels may offer the greatest benefit for enabling discontinuation. Attention should be given to how such interventions can be most effectively designed and operationalised in practice, in different healthcare systems, and how they might be integrated within existing, wider deprescribing frameworks to support uptake and sustainability.

## Supplementary Information

Below is the link to the electronic supplementary material.

Supplementary Material 1

## Data Availability

All data generated or analysed during this study are included in this published article and its supplementary information files.

## Abbreviations

ADM: Antidepressant medication
BJGP: British Journal of General Practice
CINAHL: Cumulative Index to Nursing and Allied Health Literature
ENTREQ: Enhancing transparency in reporting the synthesis of qualitative research
EPPI-Centre: The Evidence for Policy and Practice Information and Co-ordinating Centre based in the Social Science Research Unit in the Department of Social Science, Institute of Education, University College London
EPPI-Reviewer: A specialised software for different types of literature review, including systematic reviews, developed and maintained by the EPPI-Centre
EPPI-Reviewer Web: A web-based version of EPPI-Reviewer
HP: Health professional
MeSH: Medical Subject Headings thesaurus; controlled and hierarchically organised vocabulary produced by the National Library of Medicine (US)
NDLTD: Networked Digital Library of Theses and Dissertations
PRISMA: Preferred Reporting Items for Systematic Reviews and Meta-Analyses
QA: Quality appraisal
QES: Qualitative Evidence Synthesis
SEM: Bronfenbrenner’s (1977) Social Ecological Model
SNRI: Serotonin-Norepinephrine Reuptake Inhibitor
SPIDER: Sample, Phenomenon of Interest, Design, Evaluation and Research type
SR: Systematic review
SSRI: Selective Serotonin Reuptake Inhibitor
TCA: Tricyclic Antidepressant
UK: United Kingdom
US: United States
