# Supplementary material for: Discontinuing antidepressant medication: a qualitative evidence synthesis and logic model based on health professionals’ views
Source: BMC Health Serv Res. 2025 Sep 30;25:1226. doi: 10.1186/s12913-025-13445-7 (PMC12482417; doi:10.1186/s12913-025-13445-7)
Supplement: Supplementary file 1 — Supplementary Material 1 [file 12913_2025_13445_MOESM1_ESM.docx]

# Additional File 1

**List of Appendices**

[Appendix A. Search queries for PubMed, CINAHL, PsycInfo and the NDLTD. 1](#_Toc203568969)

[Appendix B. Search queries for Google Scholar and the BJGP. 6](#_Toc203568970)

[Appendix C. Quality and Relevance appraisal tool. 7](#_Toc203568971)

[Appendix D. Algorithm used to calculate the overarching ‘Usefulness’ of included studies. 10](#_Toc203568972)

[Appendix E. PRISMA 2020 27-item checklist and 12-item abstract checklist applied to this review. 11](#_Toc203568973)

[Appendix F. ENTREQ checklist applied to this review. 15](#_Toc203568974)

[Appendix G. Characteristics of included studies ordered by date of publication. 18](#_Toc203568976)

[Appendix H. Summary of findings of included studies ordered by date of publication. 23](#_Toc203568977)

[Appendix I. Thematic synthesis findings: analytical and descriptive themes. 37](#_Toc203568978)

[Appendix J. Descriptive themes from the thematic synthesis: the contribution of each study. 40](#_Toc203568979)

# Appendices

Appendix A. Search queries for PubMed, CINAHL, PsycInfo and the NDLTD.

| **PubMed** | | | |
| --- | --- | --- | --- |
| **Search number** | **Query** | **Filters** | **Results** |
| **1** | deprescri* OR de prescri* OR de-prescri* OR inappropriate prescribing OR discontinu* OR continu* OR cessation* OR cease OR dropout OR drop out OR interrupt* OR taper* OR reduc* OR withdraw* OR drug holiday OR stop* OR terminat* OR stop taking OR stopping treatment |  | 5,493,466 |
| **2** | antidepressive agents [MeSH Terms] |  | 62,423 |
| **3** | neurotransmitter uptake inhibitors [MeSH Terms] |  | 30,346 |
| **4** | psychotropic drugs [MeSH Terms] |  | 169,377 |
| **5** | psychotropic* OR antidepress* OR anti depress* OR ((serotonin OR norepinephrine OR noradrenaline OR neurotransmitt* OR dopamine*) AND (uptake OR reuptake OR re-uptake)) OR noradrenerg* OR antiadrenergic OR anti adrenergic OR SSRI OR SNRI OR TCA OR tricyclic* OR tetracyclic* OR heterocyclic* |  | 665,090 |
| **6** | #2 OR #3 OR #4 OR #5 |  | 703,409 |
| **7** | #1 AND #6 |  | 189,365 |
| **8** | general practitioners [MeSH Terms] |  | 9,612 |
| **9** | health personnel [MeSH Terms] |  | 580,370 |
| **10** | psychiatrists [MeSH Terms] |  | 108,474 |
| **11** | health professional* OR GP OR GPs OR psychiatrist* OR psychologist OR doctor* OR clinician* OR physician* OR therapist* OR nurse* OR pharmacist* OR practitioner* |  | 3,276,435 |
| **12** | #8 OR #9 OR #10 OR #11 |  | 3,488,606 |
| **13** | #7 AND #12 |  | 36,337 |
| **14** | attitude to health [MeSH Terms] |  | 460,033 |
| **15** | attitude of health personnel [MeSH Terms] |  | 167,258 |
| **16** | health behavior [MeSH Terms] |  | 351,081 |
| **17** | view* OR attitude* OR experience* OR perspective* OR belief* OR behaviour* OR behavior* OR opinion* |  | 4,193,640 |
| **18** | #14 OR #15 OR #16 OR #17 |  | 4,404,700 |
| **19** | #13 AND #18 |  | 18,458 |
| **20** | qualitative OR interview* OR focus group* OR qualitative research* OR personal narrative* OR questionnaire* OR hermeneutic* OR empirical research OR grounded theory OR qualitative synthesis |  | 1,619,938 |
| **21** | #19 AND #20 |  | 2,976 |
| **22** | #19 AND #20 | from 2018/7/1 - 3000/12/12 | 555 |
| **23** | #19 AND #20 | English, from 2018/7/1 - 3000/12/12 | 529 |

Source: Original source by authors.

| **PsycInfo** | | |
| --- | --- | --- |
| **Search number** | **Query** | **Results** |
| **1** | (deprescri* or de prescri* or de-prescri* or inappropriate prescribing or discontinu* or continu* or cessation* or cease or dropout or drop out or interrupt* or taper* or reduc* or withdraw* or drug holiday or stop* or terminat* or stop taking or stopping treatment).mp. [mp=title, abstract, heading word, table of contents, key concepts, original title, tests & measures, mesh word] | 860,396 |
| **2** | exp Antidepressant Drugs/ | 40,522 |
| **3** | exp Neurotransmitter Uptake Inhibitors/ | 14,866 |
| **4** | psychopharmacology/ | 8,905 |
| **5** | (psychotropic* or antidepress* or antidepress* or ((serotonin or norepinephrine or noradrenaline or neurotransmitt* or dopamine*) and (uptake or reuptake or re-uptake)) or noradrenerg* or antiadrenergic or anti adrenergic or SSRI or SNRI or TCA or tricyclic* or tetracyclic* or heterocyclic*).mp.[mp=title, abstract, heading word, table of contents, key concepts, original title, tests & measures, mesh word] | 87,408 |
| **6** | 2 or 3 or 4 or 5 | 102,736 |
| **7** | 1 and 6 | 31,847 |
| **8** | exp General Practitioners/ | 6,174 |
| **9** | exp Health Personnel/ | 178,385 |
| **10** | exp Psychiatrists/ | 12,371 |
| **11** | (health professional* or GP or GPs or psychiatrist* or psychologist or doctor* or clinician* or physician* or therapist* or nurse* or pharmacist* or practitioner*).mp.[mp=title, abstract, heading word, table of contents, key concepts, original title, tests &measures, mesh word] | 521,221 |
| **12** | 8 or 9 or 10 or 11 | 582,442 |
| **13** | 7 and 12 | 3,991 |
| **14** | exp Health Attitudes/ | 11,175 |
| **15** | exp Health Personnel Attitudes/ | 25,786 |
| **16** | exp Health Behavior/ | 39,318 |
| **17** | (view* or attitude* or experience* or perspective* or belief* or behaviour* or behavior* or opinion*).mp. [mp=title,abstract, heading word, table of contents, key concepts, original title, tests & measures, mesh word] | 2,569,178 |
| **18** | 14 or 15 or 16 or 17 | 2,571,253 |
| **19** | 13 and 18 | 1,789 |
| **20** | (qualitative or interview* or focus group* or qualitative research* or personal narrative* or questionnaire* or hermeneutic* or empirical research or grounded theory or qualitative synthesis).mp. [mp=title, abstract, heading word, table of contents, key concepts, original title, tests & measures, mesh word] | 999,157 |
| **21** | 19 and 20 | 541 |
| **22** | Limit 21 to (english language and yr="2018 -2023") | 73 |

Source: Original source by authors.

| **Networked Digital Library of Theses and Dissertations (NDLTD)** | | |
| --- | --- | --- |
| **Query** | **Filters** | **Results** |
| (deprescri* OR de prescri* OR de-prescri* OR discontinu* OR continu* OR cessation* OR inappropriate prescribing OR cease OR dropout OR drop out OR interrupt* OR taper* OR reduc* OR drug holiday OR stop* OR withdraw* OR terminat* OR stop taking OR stopping treatment ) AND (psychotropic* or antidepress* or anti depress* or ((serotonin or norepinephrine or noradrenaline or neurotransmitt* or dopamine*) and (uptake or reuptake or re-uptake)) or noradrenerg* or antiadrenergic or anti adrenergic or SSRI or SNRI or TCA or tricyclic* or tetracyclic* or heterocyclic*) AND (health professional* OR GP OR GPs OR psychiatrist* OR psychologist OR doctor* OR clinician* OR physician* OR therapist* OR nurse* OR pharmacist* OR practitioner*) AND (view* OR attitude* OR experience* OR perspective* OR belief* OR behaviour* OR behavior* OR opinion*) AND (qualitative OR interview* OR focus group* OR qualitative research* OR personal narrative* OR questionnaire* OR hermeneutic* OR empirical research OR grounded theory OR qualitative synthesis) | Published since 2018  English language | 344 |

Source: Original source by authors.

Appendix B. Search queries for Google Scholar and the BJGP.

| **Data source** | **Query** | **Filters** | **Results** |
| --- | --- | --- | --- |
| **Google Scholar** | antidepressant deprescribing discontinuation perspectives views health professionals therapists qualitative | Published since 2018  (Sorted by relevance) | 367 |
| **BJGP** | antidepressant discontinuation | Published since 1 July 2018  (Sorted by best match) | 17 |

Source: Original source by authors.

Appendix C. Quality and Relevance appraisal tool.

QA1 – Were steps taken to strengthen rigour in the sampling?

Consider whether:

- the sampling strategy was appropriate to the questions posed in the study (for example, was the strategy well reasoned and justified)

- attempts were made to obtain a diverse sample of the population in question (think about who might have been excluded who might have had a different perspective to offer)

- characteristics of the sample critical to the understanding of the study context and findings were presented (i.e. do we know who the participants were in terms of for example, basic socio-demographics, characteristics relevant to the context of the study?)

 Yes, a fairly thorough attempt was made

 Yes, several steps were taken

 Yes, minimal few steps were taken

 Unclear

 No, not at all / Not stated / Can't tell

QA2 – Were steps taken to strengthen rigour in the data collected?

Consider whether:

- Data collection was comprehensive, flexible and/or sensitive enough to provide a complete and/or vivid and rich description of people's perspectives and experiences (for example, did the researchers spend sufficient time at the site / with participants? Did they keep 'following up'? Was more than one method of data collection used?

- Steps were taken to ensure that all participants were able and willing to contribute (for example, processes for consent see D4), language barriers, power relations between adults and children/ young people.

 Yes, a fairly thorough attempt was made

 Yes several steps were taken

 Yes, minimal few steps were taken

 Unclear

 No, not at all / Not stated / Can't tell

QA3 – Were steps taken to strengthen rigour of the analysis of data?

Consider whether:

- data analysis methods were systematic (for example, was a method described / can a method be discerned?

- diversity in perspective was explored

- the analysis was balanced in the extent to which it was guided by preconceptions or by the data

- quality analysis in terms of inter-rater reliability/agreement

- the analysis sought to rule out alternative explanations for findings (in qualitative research this could be done by, for example, searching for negative cases/exceptions, feeding back preliminary results to participants, asking a colleague to review the data, or reflexivity

 Yes, a fairly thorough attempt was made

 Yes, several steps were taken

 Yes, minimal steps were taken

 Unclear

 No, not at all / Not stated / Can't tell

QA4 – Were the findings of the study grounded in/supported by the data?

Consider whether:

- enough data are presented to show how the authors arrived at their findings

- the data presented fit the interpretation/ support the claims about patterns in data

- the data presented illuminate/illustrate the findings

- (for qualitative studies) quotes are numbered or otherwise identified and the reader can see they don't come from one or two people.

 Well grounded / supported

 Fairly well grounded / supported

 Limited grounding / support

QA5 - Please rate the findings of the study in terms of breadth and depth?

Consider whether:

- (NB it may be helpful to consider 'breadth' as the extent of description and 'depth' as the extent to which data has been transformed/analysed)

- A range of issues are covered

- The perspectives of participants are fully explored in terms of breadth (contrast of two or more perspectives) and depth (insight into a single perspective)

- richness and complexity has been portrayed (for example, variation explained, meanings illuminated)

- There has been theoretical/conceptual development

 Good / fair breadth and depth

 Good / fair depth but very little breadth

 Good / fair breadth, but little depth

 Limited breadth and depth

QA6 – Privileges participants’ perspectives/experiences?

Consider whether:

- there was a balance between open-ended and fixed response questions

- whether children were involved in designing the research

- there was a balance between the use of an a priori coding framework and induction in the analysis

- the position of the researchers (did they consider it important to listen to the perspectives of children?)

- steps were taken to assure confidentiality and put young people at ease

 Not at all

 A little

 Somewhat

 A lot

QA7 – Reliability

Guidance: Think (mainly) about the answers you have given to questions above

Using the ratings score 3 for top answer, 2 for middle answer, and 1 for bottom answer, 0 for no answer- 15-18=high, 11-14 = medium, 0-10 = low

 Low reliability

 Medium reliability

 High reliability

QA8 – Overall how relevant is the study for this review?

Please assess the relevance of the study checking answers to the following questions:

Aims, Actual sample, Sampling/recruitment/consent, Data collection, Findings

 High overall relevance

 Medium overall relevance

 Low overall relevance

QA9 – Usefulness

Guidance: Think (mainly) about the answers you have given to questions 4-6 above and consider:

- the match between the study aims and findings and the aims and purpose of the synthesis

- its conceptual depth/explanatory power

 Low usefulness (use for a study that gets low on either)

 Medium usefulness (use for a study that gets medium on both)

 High usefulness (use for a study that gets a high and a medium)

 Gold Standard (use if study is both highly relevant and high quality)

Source: Lester et al., 2019. [89]

Appendix D. Algorithm used to calculate the overarching ‘Usefulness’ of included studies.

| **Usefulness rating** | **Criteria** |
| --- | --- |
| Gold standard | A ‘High’ rating for both Reliability and Relevance |
| High | One ‘High’ and one ‘Medium’ Reliability and Relevance rating |
| Medium | A ‘Medium’ rating for both Reliability and Relevance |
| Low | A ‘Low’ rating for either Reliability or Relevance |

Source: Lester et al., 2019. [89]

Appendix E. PRISMA 2020 27-item checklist and 12-item abstract checklist applied to this review.

| **27-item PRISMA checklist**  **27-** | | | |
| --- | --- | --- | --- |
| **Section and Topic** | **Item #** | **Checklist item** | **Location where item is reported** |
| **TITLE** | | |  |
| Title | 1 | Identify the report as a systematic review. | p. 1 |
| **ABSTRACT** | | |  |
| Abstract | 2 | See the PRISMA 2020 for Abstracts checklist. | p. 1 |
| **INTRODUCTION** | | |  |
| Rationale | 3 | Describe the rationale for the review in the context of existing knowledge. | s. 1 |
| Objectives | 4 | Provide an explicit statement of the objective(s) or question(s) the review addresses. | s. 1 |
| **METHODS** | | |  |
| Eligibility criteria | 5 | Specify the inclusion and exclusion criteria for the review and how studies were grouped for the syntheses. | s. 2.1 |
| Information sources | 6 | Specify all databases, registers, websites, organisations, reference lists and other sources searched or consulted to identify studies. Specify the date when each source was last searched or consulted. | s. 2.2 |
| Search strategy | 7 | Present the full search strategies for all databases, registers and websites, including any filters and limits used. | s. 2.3 |
| Selection process | 8 | Specify the methods used to decide whether a study met the inclusion criteria of the review, including how many reviewers screened each record and each report retrieved, whether they worked independently, and if applicable, details of automation tools used in the process. | s. 2.4 |
| Data collection process | 9 | Specify the methods used to collect data from reports, including how many reviewers collected data from each report, whether they worked independently, any processes for obtaining or confirming data from study investigators, and if applicable, details of automation tools used in the process. | s. 2.5 |
| Data items | 10a | List and define all outcomes for which data were sought. Specify whether all results that were compatible with each outcome domain in each study were sought (e.g. for all measures, time points, analyses), and if not, the methods used to decide which results to collect. | s. 2.5 |
|  | 10b | List and define all other variables for which data were sought (e.g. participant and intervention characteristics, funding sources). Describe any assumptions made about any missing or unclear information. | s. 2.5 |
| Study risk of bias assessment | 11 | Specify the methods used to assess risk of bias in the included studies, including details of the tool(s) used, how many reviewers assessed each study and whether they worked independently, and if applicable, details of automation tools used in the process. | s. 2.7 |
| Effect measures | 12 | Specify for each outcome the effect measure(s) (e.g. risk ratio, mean difference) used in the synthesis or presentation of results. | N/A |
| Synthesis methods | 13a | Describe the processes used to decide which studies were eligible for each synthesis (e.g. tabulating the study intervention characteristics and comparing against the planned groups for each synthesis (item #5)). | N/A |
|  | 13b | Describe any methods required to prepare the data for presentation or synthesis, such as handling of missing summary statistics, or data conversions. | N/A |
|  | 13c | Describe any methods used to tabulate or visually display results of individual studies and syntheses. | s. 2.6 |
|  | 13d | Describe any methods used to synthesize results and provide a rationale for the choice(s). If meta-analysis was performed, describe the model(s), method(s) to identify the presence and extent of statistical heterogeneity, and software package(s) used. | s. 2.6 |
|  | 13e | Describe any methods used to explore possible causes of heterogeneity among study results (e.g. subgroup analysis, meta-regression). | N/A |
|  | 13f | Describe any sensitivity analyses conducted to assess robustness of the synthesized results. | s. 2.7 |
| Reporting bias assessment | 14 | Describe any methods used to assess risk of bias due to missing results in a synthesis (arising from reporting biases). | N/A |
| Certainty assessment | 15 | Describe any methods used to assess certainty (or confidence) in the body of evidence for an outcome. | N/A |
| **RESULTS** | | |  |
| Study selection | 16a | Describe the results of the search and selection process, from the number of records identified in the search to the number of studies included in the review, ideally using a flow diagram. | s. 3.1 |
|  | 16b | Cite studies that might appear to meet the inclusion criteria, but which were excluded, and explain why they were excluded. | s. 3.1 |
| Study characteristics | 17 | Cite each included study and present its characteristics. | s. 3.2 |
| Risk of bias in studies | 18 | Present assessments of risk of bias for each included study. | s. 3.3 |
| Results of individual studies | 19 | For all outcomes, present, for each study: (a) summary statistics for each group (where appropriate) and (b) an effect estimate and its precision (e.g. confidence/credible interval), ideally using structured tables or plots. | N/A |
| Results of syntheses | 20a | For each synthesis, briefly summarise the characteristics and risk of bias among contributing studies. | N/A |
|  | 20b | Present results of all statistical syntheses conducted. If meta-analysis was done, present for each the summary estimate and its precision (e.g. confidence/credible interval) and measures of statistical heterogeneity. If comparing groups, describe the direction of the effect. | N/A |
|  | 20c | Present results of all investigations of possible causes of heterogeneity among study results. | N/A |
|  | 20d | Present results of all sensitivity analyses conducted to assess the robustness of the synthesized results. | s. 3.5 |
| Reporting biases | 21 | Present assessments of risk of bias due to missing results (arising from reporting biases) for each synthesis assessed. | N/A |
| Certainty of evidence | 22 | Present assessments of certainty (or confidence) in the body of evidence for each outcome assessed. | N/A |
| **DISCUSSION** | | |  |
| Discussion | 23a | Provide a general interpretation of the results in the context of other evidence. | ss. 4.1, 4.3 |
|  | 23b | Discuss any limitations of the evidence included in the review. | s. 4.2 |
|  | 23c | Discuss any limitations of the review processes used. | s. 4.2 |
|  | 23d | Discuss implications of the results for practice, policy, and future research. | s. 4.4 |
| **OTHER INFORMATION** | | |  |
| Registration and protocol | 24a | Provide registration information for the review, including register name and registration number, or state that the review was not registered. | N/A |
|  | 24b | Indicate where the review protocol can be accessed, or state that a protocol was not prepared. | N/A |
|  | 24c | Describe and explain any amendments to information provided at registration or in the protocol. | N/A |
| Support | 25 | Describe sources of financial or non-financial support for the review, and the role of the funders or sponsors in the review. | N/A |
| Competing interests | 26 | Declare any competing interests of review authors. | N/A |
| Availability of data, code and other materials | 27 | Report which of the following are publicly available and where they can be found: template data collection forms; data extracted from included studies; data used for all analyses; analytic code; any other materials used in the review. | N/A |

Source: Page et al, 2021. [92]

| **12-item PRISMA abstract checklist** | | | |
| --- | --- | --- | --- |
| **Section and Topic** | **Item #** | **Checklist item** | **Reported (Yes/No)** |
| **TITLE** | | |  |
| Title | 1 | Identify the report as a systematic review. | Y |
| **BACKGROUND** | | |  |
| Objectives | 2 | Provide an explicit statement of the main objective(s) or question(s) the review addresses. | Y |
| **METHODS** | | |  |
| Eligibility criteria | 3 | Specify the inclusion and exclusion criteria for the review. | Y |
| Information sources | 4 | Specify the information sources (e.g. databases, registers) used to identify studies and the date when each was last searched. | Y |
| Risk of bias | 5 | Specify the methods used to assess risk of bias in the included studies. | Y |
| Synthesis of results | 6 | Specify the methods used to present and synthesise results. | Y |
| **RESULTS** | | |  |
| Included studies | 7 | Give the total number of included studies and participants and summarise relevant characteristics of studies. | Y |
| Synthesis of results | 8 | Present results for main outcomes, preferably indicating the number of included studies and participants for each. If meta-analysis was done, report the summary estimate and confidence/ credible interval. If comparing groups, indicate the direction of the effect (i.e. which group is favoured). | Y |
| **DISCUSSION** | | |  |
| Limitations of evidence | 9 | Provide a brief summary of the limitations of the evidence included in the review (e.g. study risk of bias, inconsistency and imprecision). | Y |
| Interpretation | 10 | Provide a general interpretation of the results and important implications. | Y |
| **OTHER** | | |  |
| Funding | 11 | Specify the primary source of funding for the review. | N/A |
| Registration | 12 | Provide the register name and registration number. | N/A |

Source: Page et al, 2021. [92]

Appendix F. ENTREQ checklist applied to this review.

| **No** | **Item** | **Guide and description** | **Location where item is reported** |
| --- | --- | --- | --- |
| **1** | Aim | State the research question the synthesis addresses. | s. 1 |
| **2** | Synthesis methodology | Identify the synthesis methodology or theoretical framework which underpins the synthesis, and describe the rationale for choice of methodology *(e.g. meta-ethnography, thematic synthesis, critical interpretive synthesis, grounded theory synthesis, realist synthesis, meta-aggregation, meta-study, framework synthesis).* | s. 2.6 |
| **3** | Approach to searching | Indicate whether the search was pre-planned (*comprehensive search strategies to seek all available studies)* or iterative (*to seek all available concepts until they theoretical saturation is achieved)*. | s. 2.3 |
| **4** | Inclusion criteria | Specify the inclusion/exclusion criteria *(e.g. in terms of population, language, year limits, type of publication, study type).* | s. 2.1 |
| **5** | Data sources | Describe the information sources used (e.g. *electronic databases (MEDLINE, EMBASE, CINAHL, psycINFO, Econlit), grey literature databases (digital thesis, policy reports), relevant organisational websites, experts, information specialists, generic web searches (Google Scholar) hand searching, reference lists)* and when the searches conducted; provide the rationale for using the data sources. | s. 2.2 |
| **6** | Electronic Search strategy | Describe the literature search *(e.g. provide electronic search strategies with population terms, clinical or health topic terms, experiential or social phenomena related terms, filters for qualitative research, and search limits)*. | s. 2.3 |
| **7** | Study screening methods | Describe the process of study screening and sifting *(e.g. title, abstract and full text review, number of independent reviewers who screened studies).* | s. 2.4 |
| **8** | Study characteristics | Present the characteristics of the included studies *(e.g. year of publication, country, population, number of participants, data collection, methodology, analysis, research questions).* | s. 3.2 |
| **9** | Study selection results | Identify the number of studies screened and provide reasons for study exclusion *(e,g, for comprehensive searching, provide numbers of studies screened and reasons for exclusion indicated in a figure/flowchart; for iterative searching describe reasons for study exclusion and inclusion based on modifications t the research question and/or contribution to theory development).* | s. 3.1 |
| **10** | Rationale for appraisal | Describe the rationale and approach used to appraise the included studies or selected findings *(e.g. assessment of conduct (validity and robustness), assessment of reporting (transparency), assessment of content and utility of the findings).* | s. 2.7 |
| **11** | Appraisal items | State the tools, frameworks and criteria used to appraise the studies or selected findings *(e.g. Existing tools: CASP, QARI, COREQ, Mays and Pope* [[25](https://bmcmedresmethodol.biomedcentral.com/articles/10.1186/1471-2288-12-181#ref-CR25)]*; reviewer developed tools; describe the domains assessed: research team, study design, data analysis and interpretations, reporting).* | s. 2.7 |
| **12** | Appraisal process | Indicate whether the appraisal was conducted independently by more than one reviewer and if consensus was required. | s. 2.7 |
| **13** | Appraisal results | Present results of the quality assessment and indicate which articles, if any, were weighted/excluded based on the assessment and give the rationale. | s. 3.3 |
| **14** | Data extraction | Indicate which sections of the primary studies were analysed and how were the data extracted from the primary studies? *(e.g. all text under the headings “results /conclusions” were extracted electronically and entered into a computer software).* | s. 2.5 |
| **15** | Software | State the computer software used, if any. | s. 2.5 |
| **16** | Number of reviewers | Identify who was involved in coding and analysis. | ss. 2.5, 2.6 |
| **17** | Coding | Describe the process for coding of data *(e.g. line by line coding to search for concepts).* | s. 2.6 |
| **18** | Study comparison | Describe how were comparisons made within and across studies *(e.g. subsequent studies were coded into pre-existing concepts, and new concepts were created when deemed necessary).* | s. 2.6 |
| **19** | Derivation of themes | Explain whether the process of deriving the themes or constructs was inductive or deductive. | s. 2.6 |
| **20** | Quotations | Provide quotations from the primary studies to illustrate themes/constructs, and identify whether the quotations were participant quotations of the author’s interpretation. | s. 3.4 |
| **21** | Synthesis output | Present rich, compelling and useful results that go beyond a summary of the primary studies (e.g. *new interpretation, models of evidence, conceptual models, analytical framework, development of a new theory or construct).* | ss. 3.4, 3.6 |

Source: Tong et al., 2012. [93]

Appendix G. Characteristics of included studies ordered by date of publication.

| **No.** | **Lead author**  **& year of publication** | **Study objective** | **Country**  **& setting** | **Type & number of HPs** | **Age & gender of HPs** | **HP practice experience**  **& urbanity of practice** | **Data collection & analysis method** |
| --- | --- | --- | --- | --- | --- | --- | --- |
| **1** | Hølmkjær et al., 2022 [94] | To develop a robust model for the deprescribing of inappropriate antidepressants in nursing home residents with neuropsychiatric symptoms | Denmark  Nursing homes | GPs (n=6)  Pharmacists (n=1)  Nursing home staff – nurses, healthcare assistants, healthcare helpers, and management staff (n=3 groups, total number not reported) | Age not reported  Gender not reported | Experience not reported  Urbanity not reported | Semi-structured interviews  Thematic analysis |
| **2** | Van Leeuwen et al., 2022 [95] | To explore GPs’ views of discontinuing long-term ADM in nursing home residents | Belgium  Nursing homes | GPs (n=20) | Age=44.6 years (mean)  Female=12  Male=8 | Experience not reported  Urban=10  Rural=10 | Semi-structured interviews  Thematic analysis |
| **3** | Bowers et al., 2021 [100] | To develop a digital intervention to support practitioners in helping patients discontinue inappropriate long-term ADM | England  Primary care | GPs (n=17)  Nurse practitioners (n=2)  Total (n =19) | Age=44.3 years (mean)  Female=11  Male=8 | Experience=4-34 years  Urbanity not reported | Semi-structured interviews  Coding tables and thematic analysis |
| **4** | Donald et al., 2021 [97] | To explore GPs’ insights about long-term  ADM prescribing and discontinuation | Australia  Primary care | GPs (n=22) | Age=47 years (mean)  Female=9  Male=12 | Experience= 5-34 years  Urban=11  Rural=11 | Semi-structured interviews  Thematic analysis |
| **5** | Kelly et al., 2021 [98] | To explore  GPs’ perceptions and experiences of discontinuing  ADM | Republic of Ireland  Primary care | GPs (n=10) | Age not reported  Female=3  Male=7 | Experience= less than 5 years - more than 25 years  Urban=6  Rural=3  Urban and rural=1 | Interviews  Thematic analysis |
| **6** | Van Leeuwen et al., 2021 [96] | To explore GPs’ views and experiences of discontinuing long-term ADM, barriers  and facilitators of discontinuation and required support | Belgium  Primary care | GPs (n=20) | Age=44.6 years (mean)  Female=12  Male=8 | Experience  not reported  Urban=10  Rural=10 | Semi-structured interviews  Thematic analysis |
| **7** | Vaswani-Bye,  2020 [104] | To explore psychiatrists’ experiences around  deprescribing ADMs in their practice | US  Setting not reported | Psychiatrists (n=10) | Age=54.2 years (mean)  Female=5  Male=5 | Experience=6-53 years  Urbanity not reported | Interviews  Discourse analysis |
| **8** | Bowers et al., 2019 [77] | To identify and explain clinician factors that shape decisions around ADM in UK primary care | England  Primary care | GPs (n=21)  GP assistants (n=4)  Nurses  (n=7)  Health team workers and  psychotherapists (n=6)  Total (n=38) | Age not reported  Female=22  Male=12  *Demographic info on 4 participants not reported | Experience=8-34 years  Urbanity not reported | Focus groups and interviews  Thematic analysis |
| **9** | Wentink et al., 2019 [106] | To identify factors that enable  the shared decision-making process about  discontinuation of ADM between long-term  users and their GPs | Netherlands  Primary and secondary mental health care | GPs (n=10)  Primary mental health nurses  (n=4)  Psychiatrists (n=10)  Psychologists (n=3)  Total (n=27) | Age not reported  Female=17  Male=10 | Experience not reported  Urbanity not reported | Brainstorming focus-group session  Concept mapping |
| **10** | Johnson et al., 2017 [103] | To explore factors influencing GPs’ use of ADM and their doses to treat depression | Scotland  Primary care | GPs (n=28) | Age=43 years (mean)  Female=14  Male=14 | Experience=10-37 years  Urbanity not reported | Semi-structured interviews  Framework analysis |
| **11** | Bosman et al., 2016 [105] | To explore the motivations of patients and  GPs causing long-term ADM use and to gain insight into possibilities to prevent  unnecessary long-term use | Netherlands  Primary care | GPs (n=26) | Age=30-64 years  Female=15  Male=11  Urban=18  Rural=8 | Experience not reported  Urbanity not reported | Semi-structured interviews  Constant comparative analysis |
| **12** | Iden et al., 2011 [99] | To explore decision-making on treatment with ADM among doctors and nurses in nursing homes | Norway  Nursing homes | GPs (n=16)  Nurses  (n=8)  Total (n=24) | Age=30-70 years  Female=18  Male=6 | Experience=1-40 years  Urbanity not reported | Focus groups  Systematic text condensation |
| **13** | Dickinson et al., 2010 [101] | To explore the attitudes of older patients and their GPs  to taking long-term ADM therapy, and their  accounts of the influences on long-term ADM  use | England  Primary care | GPs (n=10) | Age=34-60 years  Female=6  Male=4 | Experience not reported  Urbanity not reported | Semi-structured interviews  Framework analysis |
| **14** | Pollock and Grime, 2002 [102] | To contribute to a greater understanding of patient and professional concepts of depression and its treatment as a prerequisite for developing concordance in the clinical encounter | England  Primary care | GPs (n=19)  Practice counsellors (n=2)  Pharmacists (n=20)  Total (n=41) | Age=20-70 years  Female=20  Male=21 | Experience =0-50 years  Urbanity not reported | Interviews  Thematic analysis |

Dark grey shading indicates a study was rated as having Low Usefulness and light grey shading indicates

a study was rated as having Medium Usefulness for this review.

Source: Original source by authors using data from the included studies.

Appendix H. Summary of findings of included studies ordered by date of publication.

| **No.** | **Study author & year of publication** | **Summary of findings: key themes identified by study authors about ADM discontinuation/deprescribing** |
| --- | --- | --- |
| **1** | Hølmkjær et al., 2022 [94] | - **“Importance of professional qualifications”**   In comparison to other psychotropic medication, GPs and nursing home staff were more inclined to deprescribe ADM even if treatment was initiated by other specialties; experts and GPs addressed the need for tools to deprescribe ADM; lack of non-pharmacological alternatives perceived as a barrier to deprescribing; some GPs were concerned about the educational level of nursing home staff; perception that ADM doesn’t have much of an effect on people with dementia; insufficient qualification of both nursing home staff and GPs perceived as a barrier; unfamiliarity with a patient perceived as barrier   - **“Collaboration and communication”**   Experts and GPs viewed qualified working collaboration with nursing home staff and patient relatives as a facilitator to deprescribing; clear communication structure among nursing home staff, experts and GPs was emphasised as important; HPs noted that it is important to have a clear understanding among them as what to expect from deprescribing and which symptoms the nursing home staff need to be observant of as well as how to handle uncertainties; one expert suggested that using the Neuropsychiatric Inventory – nursing home version (NPI-NH) could help in deprescribing; a symptom assessment scale was considered by both nursing home staff and GPs as a facilitator to deprescribing; nursing home staff were more cautious than GPs to deprescribe; if patients appeared stable on medication, nursing home staff didn’t think it necessary to tamper too much with it   - **“Patient and relative involvement”**   GPs and nursing home staff believed that patients could not participate in a dialog concerning deprescribing in a meaningful way; nursing home staff and GPs were unsure about how useful was to involve relatives in deprescribing efforts; there was a perception among HPs that relatives were either not interested to participate or were “too” involved or protective; GPs thought that it was time-consuming to contact relatives prior to meetings and that nursing home staff should do it; GPs and nursing home staff thought that deprescribing meetings would be more feasible without having relatives’ participation as mandatory although they thought they might have relevant information to convey and that their views should be included; GPs and nursing home staff deemed it most relevant that the nursing home staff decided on when to include the relatives; medication changes should be attempted after the patients have adjusted to the nursing home setting (3-6 months after moving in) |
| **2** | Van Leeuwen et al., 2022 [95] | - **“Reluctance to rock the boat: not worth taking the risk”**   Fear of GPs that ADM discontinuation will disturb the fragile balance of older patients; assumption that living in a nursing home is depression-inducing and by continuing ADM they empathise with them; ADMs help patients cope with changed living conditions; ADMs are the only suitable solution for nursing home patients; effectiveness of ADM is difficult to evaluate especially in older people but overall, they are considered safe and effective; discontinuation of ADM has no benefits; if a patient is stable on ADM, discontinuation is not considered; medication reviewing is energy and time consuming; repeat prescriptions is the easy and comfortable option; limited alternatives to ADM is a barrier to discontinuation (due to limited time and high workload of GPs) and GPs assume most patients would resist psychotherapy   - **“It takes at least three to tango”**   Discontinuation must involve discussion with nurses and relatives; GPs receive pressure to continue ADM from nurses and relatives; GPs empathise with nurses and relatives by continuing ADM since they are the ones that “pick up the pieces”; fear of jeopardising relationship with nurses and relatives and reluctancy to question their opinion; involving others in decision-making is time and energy consuming; nursing staff and relatives may also facilitate discontinuation by providing help, support and specialised knowledge   - **“Opening the door: triggers to discontinue the AD”**   A medication review is an opportunity to discontinue ADM especially when other HPs are involved in the discussion; the more drugs a patient is on the less likely ADM discontinuation will be prioritised; familiarity with the patient can be either a barrier or a facilitator to discontinuation; severe health problems can facilitate discontinuation; dementia makes discontinuation easy |
| **3** | Bowers et al., 2021 [100] | - **“How ADvisor HP Would Be Used in Practice”**   HPs found helpful that they could dip in and out of the tool and get only the info they needed; they thought printing copies of the information helpful both for themselves but also for patients since it could make them feel ownership of the ADM discontinuation process; the tool would be a way to demonstrate patients reliable information and proof of HPs’ decision-making; sometimes HPs feel overwhelmed with different resources and tools   - “**Pitching It at the Right Level for GPs”**   Some information on the tool was too obvious and felt patronising; such tool could help as a reminder to discuss ADM deprescribing; the tool could help HPs be more aware of withdrawal symptoms and patients’ upcoming life events and act accordingly   - **“ADvisor HP is Evidence Based”**   HPs like the idea that the tool is evidence-based and included info from NICE guidelines   - “**The Need for Brevity”**   HPs thought that the tool was more reader-friendly and accessible than current guidelines; HPs felt that the tool should be more concise so that it would be more manageable to go through   - **“ADvisor HP Is Useful”**   HPs thought the tool was useful for providing information on tapering regimes and on differentiating between relapse and withdrawal symptoms |
| **4** | Donald et al., 2021 [97] | - **“Discontinuation of long-term use of antidepressants is not a simple deprescribing decision”** GPs assess patients’ preparedness to discontinue based on their mental stability and life circumstances (employment and low financial stress, social support, awareness of triggers and healthy lifestyle); GPs would be reluctant to discontinue ADM if patients didn’t want to or weren’t feeling ready; failed previous attempts to discontinue was a barrier; dose reduction was considered good enough in certain circumstances; lack of guidelines on deprescribing meant that the decision was subjective and based on intuition; strong GP-patient alliance was considered a facilitator to raise the discontinuation subject; GPs agreed that discontinuation had a lot of benefits but a few GPs also feared the risk of relapse - **“Discontinuation of long-term use of antidepressants is a journey taken together by the patient and the GP”**   A personalised strategy must be taken for each patient in collaboration with the patient; GPs prepared patients for discontinuation by bringing up gently the subject and setting a plan for discontinuation; some GPs found standardised tools helpful for assessing the effectiveness of ADM treatment; personalised gradual tapering was considered the best approach; GPs emphasised the importance of being proactive and preparing patients on how to recognise withdrawal symptoms or relapse, and warning them that discontinuation may be uncomfortable; regular reviews during and after discontinuation were considered, and engagement with psychological support was thought important;   - **“Supporting change in GPs’ prescribing practices”**   Repeat prescribing is the easy solution and there is an underlying rule than once you start ADM nobody thinks to stop it; some GPs thought it was important to point out to patients that ADM is not a panacea for all distress; there is inadequate evidence around long-term effects of ADM so GPs can’t talk to patients about this; increased education of GPs could help empower them to follow non-pharmaceutical approaches; there is lack of guidelines on deprescribing; there is an influence from the pharmaceutical industry to medication overuse and medicalisation of depression/anxiety; psychological support is not accessible; there needs to be a shift on how mental health and ADM is viewed in society – there is a need to find other ways to manage anxiety |
| **5** | Kelly et al., 2021 [98] | - **“Shared decision-making”**   Discontinuation decisions should ideally be taken in conjunction with the patient; GPs would be reluctant to question patients’ decision to continue; ADM self-discontinuation was considered a barrier because of increased risk of relapse; support from colleagues was considered important especially in cases where patients had complex mental and physical issues; if a patient was under the care of a mental health team GPs thought it was the psychiatrists’ responsibility to deal with ADM   - **“Personalised therapy”**   GPs would consider patients’ medical factors for discontinuing ADM such as patient age and severity of depression, whether they are mentally stable and have well-functioning lives (relationships, work etc.), whether they have other illness or drug addiction; GPs would also look at current and future life circumstances, and avoid discontinuation where stressful events are anticipated   - **“Medication-tapering toolkit”**   GPs developed their own tapering regimen informed by their practice experience, knowledge shared by colleagues and NICE guidelines; GPs followed a slow tapering approach while keeping patients under frequent review and emphasised the importance of non-pharmacological approaches to support discontinuation (avoiding drugs and alcohol, and taking exercise); GPs mentioned that having guidelines on deprescribing would be helpful as well as other tools such as, inbuilt prompts on prescribing software; others suggested a ban of repeat prescribing   - **“Health service factors”**   Although GPs recognised the therapeutic value of patient consultations they emphasised the importance of having referral options for psychological interventions for supporting the deprescribing process; lack of access to such interventions was a challenge as well as the difficulty encountered by GPs to contact other colleagues (such as psychiatrists); lack of continuity with a patient was considered a barrier to discontinuation; limited time of consultations and lack of frequent reviews was also considered a barrier   - **“Concerns around tapering”**   GPs fear patient relapse and withdrawal symptoms following discontinuation and the burden of responsibility for making the wrong decision |
| **6** | Van Leeuwen et al., 2021 [96] | - **“Success stories”** GPs may discontinue ADM in response to a patient health issue, depending on the risk of harm; GPs respond to patients’ desire to stop ADM; an ‘optimal moment’ or a new positive life event facilitates discontinuation – this acts as an alternative to ADM - **“Long-term antidepressants: is there a problem?”**   ADM is considered safe and effective even if it only works as a placebo; side-effects are minimal and tolerated by patients; yearly reviews are done but only to confirm patients’ remission; GPs fear of destabilising a patient and being held responsible for it; ADM discontinuation has unpredictable risks; adverse life circumstances are a barrier to GPs considering discontinuation; certain times during the year make discontinuation more difficult (e.g. autumn/winter); GPs don’t want to discuss with patients about miserable periods they have been through; routine prescribing convenient for both parties; GPs review reactively instead of proactively   - **“Discontinuation of antidepressants is not simple”**   ADM discontinuation has no benefit and gives ‘bad news’ to patients; if a patient was stable on ADM, ADM was considered as effective and necessary, and discontinuation was not prioritised; discontinuation is difficult, time and energy consuming; it is easier for GPs to discuss discontinuation in spring or summer due to lower workload; GPs do not think it is their role to discuss discontinuation and will only do so when the patients ask themselves; GPs fear jeopardising their relationship with their patients by discussing discontinuation; patient familiarity is both a barrier and a facilitator; GPs are reluctant to question decisions of patients’ previous doctors; GPs could benefit from collaboration with other HPs but psychotherapy accessibility is limited; current collaboration with other HPs is poor; lack of guidelines and tools on managing long-term ADM is a barrier to discontinuation |
| **7** | Vaswani-Bye,  2020 [104] | - **“Depression is a medical illness”**   Depression is a disease like diabetes; psychiatrists felt a duty to alleviate suffering through ADMs; they believed that ADM treats the underlying condition of depression and some patients, especially those with severe depression, need them indefinitely; some psychiatrists felt they could tell whether a patient needs ADM for long or short term; they tell patients that if discontinuation fails they can always try again later; ADM was considered by some psychiatrists as the main treatment with therapy as adjunctive; therapy was also considered helpful for supporting the discontinuation process; psychiatrists would often act paternalistic towards their patients because they felt they know more about medicines   - **“Depression is an Understandable Reaction to Difficult Life Circumstances”**   This group of psychiatrists believed that ADM is a temporary solution for alleviating certain symptoms; they tended to avoid or delay ADM as much as possible; they considered ADM as an adjunctive treatment and therapy as the primary treatment; they thought that discontinuation could be attempted with all patients; listening to patients’ stories and helping them contextualise their problems was considered key; this group of psychiatrists viewed ADM as harmful and toxic to the nerves; they liked to emphasise to patients that depression is not solely a chemical imbalance but a multi-faceted condition; psychiatrists recognised that depression was often experienced by marginalised people because of their difficult life circumstances; they questioned the observed mainstream shift towards biological psychiatry   - **“Facilitators to Deprescribing: Developing Practice-Based Evidence”**   Psychiatrists noted that they were clear with patients regarding uncertainties on how ADM work and that different patients respond to them differently; they also warned patients that with ADM there is a lot of trial and error; where patients really wanted to stay on them, psychiatrists would respect their desire; a strong therapeutic relationship with patients was a facilitator to discontinuation; mirroring patients’ language and focusing on their goals for treatment was a facilitator to discontinuation; psychiatrists thought it was important to let patients know they could reach out to them during discontinuation; collaboration with patients’ relatives and other HPs was also helpful for deprescribing; mentioning deprescribing at the initiation of treatment helped future discontinuation efforts; revisiting and discussing the idea of discontinuation throughout treatment was a facilitator; patient desire to discontinue was also considered a facilitator; observing benefits from discontinuation helped to continue with the process; psychiatrists noted that they attempted deprescribing by innovating and collating evidence (e.g. via different and personalised tapering strategies)   - **“Barriers to Deprescribing: Challenges in Implementation”**   ADMs are physiologically and psychologically addictive; sometimes full discontinuation was not possible and ADMs were instead reduced to a low dose; the type of ADM played a role in withdrawal symptoms; lack of evidence in deprescribing was considered a barrier to discontinuation; interests of Big Pharma and commodification of health disincentivised deprescribing; a cultural overreliance on treating mental health problems through medication was also viewed as a barrier to deprescribing |
| **8** | Bowers et al., 2019 [77] | - **“Who is responsible for broaching the subject of discontinuation?”**   Some HPs said it was the patients’ responsibility to broach the subject of discontinuation and that this expectation should be set at ADM initiation; HPs assumed that if patients didn’t say anything it meant that they wanted to continue ADM but also acknowledged that they are often hesitant to talk about discontinuation; HPs say that continuing ADM is easier than raising the subject of discontinuation; some HPs said it was the person who prescribes ADM who is responsible for deprescribing but being proactive is difficult; other HPs suggested that the decision needs to be in collaboration with the patient and that support from other HPs can also be helpful   - **“Risk of destabilising current situation”**   HPs fear that they may destabilise a patient that is doing well and assumed that patients also wouldn’t want to risk their current situation if they were feeling well   - **“Continuity and knowing the patient makes it easier to discuss discontinuation”**   Mentioning discontinuation at ADM initiation was perceived a facilitator; familiarity with patients’ circumstances and medical history and experience was also a facilitator   - **“A HP’s confidence in their skills and knowledge”**   Lack of skills and unclear, inaccessible and inapplicable guidelines on deprescribing was considered a barrier to discontinuation; HPs were unfamiliar with or did not use NICE guidelines   - **“Organisational barriers and enablers to discussing discontinuation”**   Patients seeing different practitioners (lack of continuity) was considered a barrier; time constraints (10 minute consultations) were also a barrier; computer system tools which prompt medication reviews would be useful in successful discontinuation |
| **9** | Wentink et al., 2019 [106] | - **“Process of discontinuation”** HPs prioritised topics such as the ‘Provision of information in relation to withdrawal symptoms’, ‘Prevention of relapse’, ‘Individual dose-reduction schedule’ and ‘Fear of relapse’ - **“Expectations”**   HPs prioritised topics such as ‘Previous experiences with dose reduction’ and ‘Expectations: what do you hope to achieve?’   - **“Professional guidance”**   HPs prioritised topics such as ‘Regular counselling sessions to provide guidance (check-ups)’ and ‘How quickly can the person providing counselling be contacted?’   - **“Current use”**   HPs prioritised topics such as ‘Current effect. What effect do you notice of the medicine (that is, calm, balanced, flat) and what do you think of that?’, ‘Reason(s) to stop taking antidepressants’ and ‘Current side effects of medication’   - **“Environment”**   HPs prioritised topics such as ‘Involve partner and social environment’ and ‘Ensure stable life circumstances during reduction (no life event)’   - **“Side effects”**   HPs prioritised topics such as ‘Suicidality’ and ‘Current quality of life and psychological functioning’ |
| **10** | Johnson et al., 2017 [103] | - **“Depression diagnosis and management”**   GPs looked at the person as a whole to make a diagnosis and valued the therapeutic role of their consultations; they would adopt a ‘watch and wait’ approach to see whether depression symptoms would go away; GPs often felt there was an expectation/pressure from patients to be prescribed ADM; in cases of more severe depression symptoms GPs might prescribe at the first presentation; GPs thought that ADM should not be the only treatment for depression but also embraced and supported other alternative options   - **“Patients’ expectations and characteristics”**   Time pressures discouraged the use of non-pharmacological alternatives; GPs said that ADM was viewed as a ‘quick fix’ for some patients   - **“GPs’ experience and relationships”**   GPs prescribed ADM based on a mixture of formal training and informal reflective practice but as they gained experience they also became more ‘idiosyncratic’ as to the best ways to manage depression; national and local guidelines were considered by GPs to weakly influence prescribing but local prescribing resources, such as the formulary and prescribing support teams, did influence drug choices and cost effective prescribing decisions   - **“Antidepressant use: safety, risk management and effectiveness”**   Some kind of ADM was considered more safe than others and GPs differentiated side-effects among different ADMs; the majority of GPs considered higher SSRI doses were more efficacious; psychiatrists routinely ‘pushed the dose’ of SSRIs; some prescribers said they increased doses as a response to patients’ expectation to do something   - **“Review frequency”**   Reactive rather than proactive reviews meant that ADM may not be reduced when it is appropriate and GPs not feeling comfortable to reduce or stop the medication at a later date; the Medicines and Healthcare products Regulatory Agency (MHRA) citalopram warning was considered to have facilitated proactive reviews leading in ADM reduction; fear of relapse, especially for patients with chronic depression, was considered a barrier to reduce/stop ADM; GPs also assumed that a lot of patients are psychologically dependent on ADM and resisted reductions |
| **11** | Bosman et al., 2016 [105] | - **“Patient-GP dyads”**   GPs felt confident to help patients discontinue ADM but patients did not necessarily agree; GPs thought the consultation time was enough whereas patients needed more time; policies around medication review were varied and inconsistent; it was unclear who is responsible for initiating discussion on ADM discontinuation; GPs said that when patients were stable there was no need to discontinue and that they should be the ones to contact them should their situation changes or when they want to discontinue   - **“Supportive guidance”**   GPs acknowledged that GPs themselves, mental health assistants, psychiatrists or psychologists should provide supportive guidance on discontinuation and that patient relatives can also help with monitoring and provide supportive guidance; GPs indicated that more specific guidelines on discontinuation are needed; support after discontinuation is important; continuation can be a result of ignorance, neglect, and increased workload of GPs; automatic warning on repeat prescriptions could be useful for discontinuation   - **“Personal circumstances”**   Patient’s improved functioning was both a barrier and a facilitator to ADM discontinuation; ADM side-effects were also considered an important factor for discontinuation; GPs indicated that for successful discontinuation, past relapses should be limited and patients should have faith/confidence in themselves that they were able to discontinue   - **“Patient-GP considerations”**   A belief that ADM cures a chemical imbalance was a barrier to ADM discontinuation although a few GPs described ADM as chemical/unnatural; GPs also assumed that most patients are psychologically dependent on ADM; nearly half of GPs said that patients are better off without medication; fear of relapse was a barrier to discontinuation; GPs noticed that symptoms were only present during winter so ADM should be discontinued during other seasons |
| **12** | Iden et al., 2011 [99] | - **“Depressed or just tired of life?”**   Doctors found diagnosing depression unclear and based their conclusions on nurses’ observations; nurses were not skilled in diagnosing depression; limited time of GPs mean less time spent in making accurate diagnoses   - **“To treat or not to treat with antidepressants?”**   Given time constraints of nursing staff, ADM was considered the simplest solution; there were differing opinions on whether sorrow warrants ADM; unpredictable withdrawal symptoms and fear of destabilising patients was a barrier to discontinuation; there is lack of frequent reviews and doctors rely on nurses’ observations instead; financial constraints, conservative treatment traditions and staff attitudes were a barrier to ADM alternatives   - **“Who determines the treatment?”**   Unskilled and auxiliary nursing personnel often ask for patients to be treated with ADM; doctors were reluctant to question nurses’ observations; GPs often felt pressure by nurses to continue prescribing ADM since it would make patients more aggressive and resist care |
| **13** | Dickinson et al., 2010 [101] | - **“The benefits of ADM”**   ADM was viewed as effective for alleviating distressing symptoms, irrespective of their precise action (placebo or otherwise); prescribing ADM is a way for doctors to show they are doing something; a lot of social factors of depression cannot be solved; ADM is cheap and effective   - **“Ambiguities and dissonances in the understanding of depression and its treatment”**   Diagnosing depression is often subjective; some GPs view depression as a medical condition; psychological support is often inaccessible so ADM is the best suitable option; GPs assumed that elderly patients resist to engage with psychotherapy; some GPs thought that ADM are often prescribed as ‘lifestyle drugs’ and perhaps this should not be the case   - **“Barriers to the discontinuation of ADM”**   Pre-warning patients of the duration of ADM treatment, tapering the dose and scheduling discontinuation around spring were considered facilitators to discontinuation; where GPs had limited confidence in their persuasive power discontinuation was more difficult; extensive ADM treatment and lengthy medical history of patients was a barrier to discontinuation; GPs thought that for many patients they cannot change a lot of the factors that caused depression; ADM was seen as validating the illness and sending a message to the family; there is often an expectation from patients to be prescribed an ADM; in elderly patients, GPs felt they had more important issues to deal with (other health issues) than to consider ADM deprescribing; fear of destabilising the patient and reluctancy to change the status quo was a barrier to discontinuation; GPs generally left it to the patient to decide whether or not to discontinue but in cases where this was considered necessary, GPs felt they could manage deprescribing especially with the support of other HPs |
| **14** | Pollock and Grime, 2002 [102] | - **“Time and Concordance in the Consultation”***   GPs considered ADM as safe and effective, that they had sufficient skills for providing counselling support and that they could use time flexibly according to patients’ specific needs; ADM was seen as the primary treatment and counselling as useful adjunct; ADM was considered to work more quickly than therapy and was readily available   - **“Antidepressants”**   There was an acknowledgement that ADM does not treat the cause of depression but may indirectly help patients deal with their problems through improved mood; other GPs believed that ADM cures a chemical imbalance in the brain; GPs observed an inverse relationship between giving consultation time and prescribing ADM; GPs would honour patients’ desire to stop ADM but they would insist continuing if they knew patients anticipated stressful periods; GPs would not stop ADM around Christmas; GPs were unaware of the difficulties patients experienced in taking ADM and their fear and disappointment of relapsing after discontinuation; self-discontinuation was expected by GPs to often result in relapse; although some GPs advised a gradual discontinuation, some felt that ADM half-life was sufficient to enable sudden discontinuation; GPs said it was important to explain to patients that if discontinuation fails they can try again at another time; GPs believed that many patients were psychologically dependent on ADM; there was a general rule among GPs that ADM should be taken for at least 6 months and 3-4 months after recovery; GPs encouraged patients to reach out to them immediately if they felt bad following discontinuation   - **“Community Pharmacists and Depression”**   Community pharmacists had low confidence in their knowledge of depression and its treatment; pharmacists felt they were more accessible than GPs for discussing depression and its treatment; pharmacists said they felt lack of time and privacy in a busy pharmacy setting to be able to discuss depression and ADM; they were often working in isolation from other HPs; they were reluctant to question prescribers’ treatment decisions; they were not always sure whether they should discuss ADM side-effects with patients; they felt that in most circumstances they could assess whether ADM alternatives are appropriate; they considered it important to warn patients of ADM’s delay in therapeutic effect and monitor patients’ treatment (change in medication or dosage, repeat prescriptions)  *This study did not employ a typical IMRaD (Introduction, Methods, Results, and Discussion) structure. Relevant findings found across the study were extracted and grouped under three chapter headings (not themes). |

Dark grey shading indicates a study was rated as having Low Usefulness and light grey shading indicates

a study was rated as having Medium Usefulness for this review.

Source: Original source by authors using data from the included studies.

Appendix I. Thematic synthesis findings: analytical and descriptive themes.

| **Analytical themes** | **Descriptive themes** | |
| --- | --- | --- |
|  | **Barriers** | **Facilitators** |
| **HPs’ Perceptions of**  **ADM** | - Safe and effective - Primary treatment - Discontinuation bears no benefits | - Addictive/unnatural and uncertain efficacy - Adjunctive treatment - Discontinuation has benefits (e.g. increase of personal agency, investment in psychological change and cessation of side-effects) |
| **HPs’ Perceptions of**  **Depression** | - Chronic, medical illness | - Circumstantial reaction/multifactorial problem |
| **HPs’**  **Sense of**  **Professional Duty** | - Unethical to withhold ADM treatment/must alleviate suffering - Validating the illness and giving hope through ADM - Belief that it is the patients' responsibility to initiate ADM discontinuation discussion - Not questioning patients' and other HPs' decision to continue ADM - Maintaining patient situation via ADM | - Investing in therapeutic relationship and building on patients' strengths - Belief that it is the prescribers’ responsibility to initiate ADM discontinuation discussion - Improving patient situation through ADM discontinuation |
| **HPs’**  **Confidence in Supporting ADM Discontinuation** | - Fears and insecurities (e.g. of destabilising patient, being held accountable, jeopardising relationships with patients/relatives/other HPs, dealing with uncomfortable situations) - Excessive confidence in ADM leading to paternalism | - Being transparent about medical uncertainty and ADM risks - Embracing a 'trial and error' journey of discontinuation |
| **HPs’ Assessment of Patients' Circumstances and**  **Characteristics** | - Old age, disabled and/or living in a nursing home - Drug/alcohol addiction or polypharmacy - Unfavourable ADM treatment history (e.g. failed discontinuation attempts, long treatment duration) - Family history of depression - Severe/recurring depression | - Strong patient support network - Stability (e.g. mental, financial, relationship, no upcoming major events) - Engagement in self-care and healthy lifestyle - Positive life events - Health issues (including serious ADM side-effects, pregnancy and dementia) - Mild/moderate depression |
| **HPs’ Assessment of Patients’ Desires, Motivations**  **and Capabilities** | - Patient is psychologically dependent on ADM - Patient wishes to continue ADM - Patient not motivated/able to follow ADM alternatives - Patient not interested in their medication treatments | - Patient desires/feels capable to stop ADM - Patient is aware of depression/anxiety triggers |
| **Systemic Healthcare Delivery Issues** | - HP unavailability/   inaccessibility (due to factors such as, lack of time or privacy)   - Inaccessible or limited ADM alternatives - Lack of patient continuity - Inaccessible, unclear or insufficient knowledge on depression and ADM - Lack of relevant training/tools - Poor collaboration among HPs/professional hierarchy | - Personalised tapering techniques (e.g. experimenting with ADM dosage, avoiding discontinuation during winter) - Receiving support and collaborative decision-making (involving other HPs, patients or patients’ relatives) - Planning treatment duration and setting goals/expectations at ADM initiation |
| **Societal Norms and Pressures** | - Growing emphasis on biological psychiatry and development of drug treatments - Repeat prescribing as an underlying rule among HPs/lack of medication reviews - Expectation/pressure from patients, patients’ relatives or other HPs to prescribe/continue ADM | - Awareness/acknowledgment of overreliance on medication |

Source: Original work by authors.

Appendix J. Descriptive themes from the thematic synthesis: the contribution of each study.

| **Descriptive themes** | **Hølmkjær et al., 2022 [94]** | **Van Leeuwen et al., 2022 [95]** | **Bowers et al., 2021 [100]** | **Donald et al., 2021 [97]** | **Kelly et al., 2021 [98]** | **Van Leeuwen et al., 2021 [96]** | **Vaswani-Bye, 2020 [104]** | **Bowers et al., 2019 [77]** | **Wentink et al., 2019 [106]** | **Johnson et al., 2017 [103]** | **Bosman et al., 2016 [105]** | **Iden et al., 2011 [99]** | **Dickinson et al., 2010 [101]** | **Pollock and Grime, 2002 [102]** |
| --- | --- | --- | --- | --- | --- | --- | --- | --- | --- | --- | --- | --- | --- | --- |
| Analytical Theme 1: **HPs’ Perceptions of ADM** | | | | | | | | | | | | | | |
| (B) Safe and effective |  | **✓** |  |  |  | **✓** | **✓** |  |  | **✓** |  |  | **✓** | **✓** |
| (B) Primary treatment |  | **✓** |  | **✓** |  |  | **✓** |  |  | **✓** |  |  | **✓** | **✓** |
| (B) Discontinuation bears no benefits |  | **✓** |  |  |  | **✓** |  |  |  |  |  |  |  |  |
| (F) Addictive, toxic/unnatural and uncertain efficacy |  |  |  |  |  | **✓** | **✓** |  |  |  | **✓** |  |  | **✓** |
| (F) Adjunctive treatment |  |  |  |  |  |  | **✓** |  |  | **✓** |  |  |  |  |
| (F) Discontinuation has benefits (e.g. increase of personal agency, investment in psychological change and cessation of side-effects) |  |  |  | **✓** |  |  | **✓** |  |  |  |  |  |  |  |
| Analytical Theme 2: **HPs’ Perceptions of Depression** | | | | | | | | | | | | | | |
| (B) Chronic, medical illness |  | **✓** |  |  |  |  | **✓** |  |  |  | **✓** | **✓** | **✓** | **✓** |
| (F) Circumstantial reaction/multifactorial problem |  |  |  | **✓** |  |  | **✓** |  |  |  |  | **✓** | **✓** | **✓** |
| Analytical Theme 3: **HPs’ Sense of Professional Duty** | | | | | | | | | | | | | | |
| (B) Unethical to withhold ADM treatment/must alleviate suffering |  | **✓** |  | **✓** |  | **✓** | **✓** |  |  | **✓** |  |  | **✓** |  |
| (B) Validating the illness and giving hope through ADM |  |  |  |  |  |  | **✓** |  |  | **✓** |  |  | **✓** |  |
| (B) Belief that it is the patients' responsibility to initiate ADM discontinuation discussion |  | **✓** | **✓** |  | **✓** | **✓** |  | **✓** |  |  | **✓** |  |  |  |
| (B) Not questioning patients' and other HPs' decision to continue ADM | **✓** | **✓** |  | **✓** | **✓** | **✓** | **✓** |  |  |  |  | **✓** |  | **✓** |
| (B) Maintaining patient situation via ADM | **✓** | **✓** | **✓** | **✓** |  |  |  | **✓** |  |  | **✓** | **✓** |  |  |
| (F) Investing in therapeutic relationship and building on patients’ strengths |  | **✓** |  | **✓** | **✓** |  | **✓** |  | **✓** | **✓** |  |  |  | **✓** |
| (F) Belief that it is the prescribers’ responsibility to initiate ADM discontinuation discussion |  |  |  |  |  |  |  | **✓** |  |  |  |  |  |  |
| (F) Improving patient situation through ADM discontinuation |  | **✓** |  | **✓** |  |  |  |  |  |  |  |  |  |  |
| Analytical Theme 4: **HPs’ Confidence in Supporting ADM Discontinuation** | | | | | | | | | | | | | | |
| (B) Fears and insecurities (e.g. of destabilising patient, being held accountable, jeopardising relationships with patients/relatives/other HPs, dealing with uncomfortable situations) | **✓** | **✓** |  | **✓** | **✓** | **✓** | **✓** | **✓** | **✓** | **✓** | **✓** | **✓** | **✓** |  |
| (B) Excessive confidence in ADM leading to paternalism |  |  |  |  |  |  | **✓** |  |  | **✓** |  |  |  |  |
| (F) Being transparent about medical uncertainty and ADM risks | **✓** |  |  | **✓** |  |  | **✓** |  | **✓** |  |  |  |  | **✓** |
| (F) Embracing a 'trial and error' journey of discontinuation |  |  |  |  |  |  | **✓** |  | **✓** |  |  |  |  | **✓** |
| Analytical Theme 5: **HPs’ Assessment of Patients’ Circumstances and Characteristics** | | | | | | | | | | | | | | |
| (B) Old age, disabled and/or living in a nursing home | **✓** |  |  | **✓** | **✓** | **✓** |  |  |  |  |  | **✓** | **✓** |  |
| (B) Drug/alcohol addiction or polypharmacy |  |  |  |  | **✓** | **✓** | **✓** |  | **✓** |  |  |  |  |  |
| (B) Unfavourable ADM treatment history (e.g. failed discontinuation attempts, long treatment duration) |  |  |  | **✓** | **✓** |  | **✓** |  | **✓** | **✓** |  |  | **✓** | **✓** |
| (B) Family history of depression |  |  |  |  |  |  | **✓** |  | **✓** |  |  |  |  |  |
| (B) Severe/recurring depression |  |  |  |  | **✓** |  | **✓** |  | **✓** | **✓** |  |  | **✓** |  |
| (F) Strong patient support network |  |  |  |  | **✓** |  |  | **✓** | **✓** |  |  |  |  |  |
| (F) Stability (e.g. mental, financial, relationship, no upcoming major events) |  |  |  | **✓** | **✓** |  |  |  | **✓** |  |  |  |  | **✓** |
| (F) Engagement in self-care and healthy lifestyle |  |  |  | **✓** | **✓** |  |  |  |  |  |  |  |  |  |
| (F) Positive life events |  | **✓** |  |  |  | **✓** |  |  |  |  |  |  |  |  |
| (F) Health issues (including serious ADM side-effects, pregnancy and dementia) | **✓** | **✓** |  |  |  | **✓** |  |  | **✓** |  | **✓** |  |  |  |
| (F) Mild/moderate depression |  |  |  |  | **✓** |  | **✓** |  |  | **✓** |  |  |  |  |
| Analytical Theme 6: **HPs’ Assessment of Patients’ Desires, Motivations and Capabilities** | | | | | | | | | | | | | | |
| (B) Patient wishes to continue ADM |  |  |  | **✓** |  |  | **✓** |  |  | **✓** |  |  | **✓** | **✓** |
| (B) Patient wishes to continue ADM |  | **✓** | **✓** |  |  |  |  | **✓** |  |  |  |  |  |  |
| (B) Patient not motivated/able to follow ADM alternatives |  | **✓** |  |  |  | **✓** |  |  |  |  |  |  | **✓** |  |
| (B) Patient not interested in their medication treatments |  | **✓** |  |  |  |  |  |  |  |  |  |  |  |  |
| (F) Patient desires/feels capable to stop ADM |  | **✓** |  | **✓** |  |  | **✓** |  |  |  | **✓** |  | **✓** | **✓** |
| (F) Patient is aware of depression/anxiety triggers |  |  |  | **✓** |  |  |  |  |  |  |  |  |  |  |
| Analytical Theme 7: **Systemic Healthcare Delivery Issues** | | | | | | | | | | | | | | |
| (B) HP unavailability/inaccessibility (due to factors such as, lack of time or privacy) | **✓** | **✓** | **✓** | **✓** | **✓** | **✓** |  | **✓** |  | **✓** | **✓** | **✓** |  | **✓** |
| (B) Inaccessible or limited ADM alternatives | **✓** | **✓** |  | **✓** | **✓** | **✓** |  |  |  |  |  | **✓** | **✓** |  |
| (B) Lack of patient continuity | **✓** |  |  |  | **✓** | **✓** | **✓** | **✓** |  |  | **✓** |  |  |  |
| (B) Inaccessible, unclear or insufficient knowledge on depression and ADM |  | **✓** | **✓** | **✓** | **✓** | **✓** | **✓** | **✓** |  |  | **✓** | **✓** | **✓** | **✓** |
| (B) Lack of relevant training/tools | **✓** | **✓** | **✓** | **✓** | **✓** | **✓** |  | **✓** |  |  | **✓** | **✓** |  |  |
| (B) Poor collaboration among HPs/professional hierarchy | **✓** | **✓** |  |  | **✓** | **✓** | **✓** |  |  |  |  |  |  | **✓** |
| (F) Personalised tapering techniques (e.g. experimenting with ADM dosage, avoiding discontinuation during winter) |  | **✓** |  | **✓** | **✓** |  | **✓** |  | **✓** | **✓** | **✓** |  | **✓** | **✓** |
| (F) Receiving support and collaborative decision-making (involving other HPs, patients or patients’ relatives) | **✓** | **✓** |  | **✓** | **✓** | **✓** | **✓** | **✓** | **✓** |  | **✓** |  | **✓** | **✓** |
| (F) Planning treatment duration and setting goals/expectations at ADM initiation |  | **✓** |  | **✓** |  |  | **✓** | **✓** | **✓** |  |  |  | **✓** |  |
| Analytical Theme 8: **Societal Norms and Pressures** | | | | | | | | | | | | | | |
| (B) Growing emphasis on biological psychiatry and development of drug treatments |  |  |  | **✓** |  |  | **✓** |  |  | **✓** |  |  |  |  |
| (B) Repeat prescribing as an underlying rule among HPs/lack of medication reviews |  | **✓** |  | **✓** |  | **✓** | **✓** | **✓** |  |  | **✓** |  |  |  |
| (B) Expectation/pressure from patients, patients’ relatives and other HPs to prescribe/continue ADM | **✓** | **✓** |  |  |  | **✓** | **✓** |  |  | **✓** |  | **✓** | **✓** |  |
| (F) Awareness/acknowledgment of overreliance on medication |  |  |  | **✓** |  |  | **✓** |  |  | **✓** |  |  | **✓** |  |

Dark grey shading indicates a study was rated as having Low Usefulness and light grey shading indicates a study was rated as having Medium Usefulness for this review. (B) indicates the descriptive theme is a barrier; (F) indicates the descriptive theme is a facilitator.

Source: Original source by authors.
